# Mutagenesis and functional analysis of SotB: A multidrug transporter of the major facilitator superfamily from *Escherichia coli*

**DOI:** 10.3389/fmicb.2022.1024639

**Published:** 2022-10-28

**Authors:** Guoqing Zhai, Zhengyu Zhang, Changjiang Dong

**Affiliations:** Department of Endocrinology, Zhongnan Hospital of Wuhan University, School of Pharmaceutical Sciences, Wuhan University, Wuhan, China

**Keywords:** MFS transporters, antiporters, multidrug resistance, transport mechanism, radiotracer, surface plasmon resonance, molecular docking, AlphaFold 2

## Abstract

Dysfunction of the major facilitator superfamily multidrug (MFS Mdr) transporters can lead to a variety of serious diseases in human. In bacteria, such membrane proteins are often associated with bacterial resistance. However, as one of the MFS Mdr transporters, the physiological function of SotB from *Escherichia coli* is poorly understood to date. To better understand the function and mechanism of SotB, a systematic study on this MFS Mdr transporter was carried out. In this study, SotB was found to directly efflux L-arabinose in *E. coli* by overexpressing *sotB* gene combined with cell based radiotracer uptake assay. Besides, the surface plasmon resonance (SPR) studies, the L-arabinose inhibition assays, together with precise molecular docking analysis, reveal the following: (i) the functional importance of E29 (protonation), H115/N343 (substrate recognition), and W119/S339 (substrate efflux) in the SotB mediated export of L-arabinose, and (ii) for the first time find that D-xylose, an isomer of L-arabinose, likely hinders the binding of L-arabinose with SotB as a competitive inhibitor. Finally, by analyzing the structure of SotB2 (shares 62.8% sequence similarity with SotB) predicted by AlphaFold 2, the different molecular mechanism of substrate recognition between SotB and SotB2 is explained. To our knowledge, this is the first systematic study of MFS Mdr transporter SotB. The structural information, together with the biochemical inspections in this study, provide a valuable framework for further deciphering the functional mechanisms of the physiologically important L-arabinose transporter SotB and its family.

## Introduction

Living cells need to continuously absorb nutrients and expel metabolic waste or toxic substances to maintain their normal growth, and this process usually requires transport proteins (Jiang et al., 2013). The Transporter Classification Database (TCDB)^1^ classifies all the transport proteins into 16 families based on their phylogeny and function, and the major facilitator superfamily (MFS family) is one of them (Saier et al., 2016). The MFS family is the largest known superfamily of secondary active transporters (Pao et al., 1998). This family is extremely widespread, and their main function in bacteria and humans is to absorb nutrients and excrete harmful substances, therefore, MFS proteins play an important role in the growth, metabolism and other normal physiological activities of organisms (Cura and Carruthers, 2012; Smith et al., 2013; Quistgaard et al., 2016).

Structural biology studies have shown that MFS proteins usually contain two domains, N-terminal domain and C-terminal domain, and each domain usually contains 6 transmembrane helixes (TMs) connected by a long and flexible intracellular loop (Pao et al., 1998; Abramson et al., 2003; Huang et al., 2003; Reddy et al., 2012). MFS proteins can be divided into symporter, antiporter and uniporter groups, depending on if the ion and substrate are moving in same or opposite directions (Heng et al., 2015). For the MFS symporters, the substrate and coupled ion(s) have the same transport direction. For the MFS antiporters, the substrate and coupled ion(s) are in opposite transport direction. Uniporters, on the other hand, do not need ion(s) in the process of substrate transport (Yan, 2013; Quistgaard et al., 2016). Both symporters and antiporters energize their transport by utilizing the proton motive force (PMF), also known as the proton electrochemical gradient (ΔμH^+^), which is made up of the proton concentration difference (ΔpH) and the membrane potential (Δψ; Yan, 2015).

Multidrug (Mdr) transporters are integral membrane proteins that use ATP or ion electrochemical gradients for actively removing chemically and structurally dissimilar toxic compounds from the cell. Mdr transporters exist in all kingdoms of life and often induce Mdr resistance, thus posing major health problems (Higgins, 2007; Li and Nikaido, 2009). Mdr transporters can be divided into five families, of which the MFS family is the largest (Kuroda and Tsuchiya, 2009). The most widespread and understood microbial MFS Mdr transport systems belong to the 12-TMs drug:H^+^ antiporter 1 (DHA12) and 14-TMs drug:H^+^ antiporter 2 (DHA14) families (Paulsen and Skurray, 1993; Saier and Paulsen, 2001). MFS Mdr transporters are antiporters, which recognize and extrude a large range of structurally unrelated drugs from the cell using the free energy released from the downhill flux of ions along their electrochemical gradient (Lewinson et al., 2006; Fluman and Bibi, 2009; Tirosh et al., 2012). MFS Mdr transporters are found across all kingdoms of life, but are most highly represented among microbial genomes, where they render cells resistant to multiple drugs (Nishino and Yamaguchi, 2001; Pasqua et al., 2019). Of course, MFS Mdr transporters do not only expel drugs, but also can transport other specific and physiologically relevant substrates like sugars.

Although MFS Mdr transporters play important roles in cellular physiology and drug development, the mechanism of drug efflux for MFS Mdr transporters is still lacking of systematic understanding (Chitsaz and Brown, 2017). So far, only several MFS Mdr antiporters of the DHA12 family have been characterized structurally from *Escherichia coli*, namely EmrD, YajR, MdfA, SotB, and LmrP from *Lactococcus lactis* (Yin et al., 2006; Jiang et al., 2013; Heng et al., 2015; Liu et al., 2016; Nagarathinam et al., 2018; Zomot et al., 2018; Wu et al., 2019; Debruycker et al., 2020; Wu et al., 2020; Xiao et al., 2021). More recently, the first DHA14 family Mdr transporter NorC from *Staphylococcus aureus* is determined, which in contrast to DHA12 members have 14-TMs (Kumar et al., 2020). MdfA and LmrP are biochemically well-characterized, while the functional analysis of YajR, EmrD, NorC, and SotB is less well-known.

The SotB protein (TCDB ID: 2.A.1.2.15) from *E. coli* has been classified as a H^+^ dependent MFS Mdr transporter. Previous studies have shown that SotB is responsible for the efflux of IPTG and L-arabinose in *E. coli* (Bost et al., 1999; Carole et al., 1999; Koita and Rao, 2012). Despite the structures of apo SotB and SotB-IPTG complex from *E. coli* has been solved by Xiao *et al* (Xiao et al., 2021), the physiological function, transport mechanism and structures of different transport states of SotB/SotB-L-arabinose complex are still poorly understood so far. Moreover, unlike *Erwinia chrysanthemi*-derived SotB2 (shares 62.8% sequence similarity with SotB), which can transport disaccharides such as melibiose, so far, no studies have shown that SotB can transport disaccharides. Although the two transporters have high homology, the transport substrates of them are quite different, and the structural basis of this difference has not yet been explained. This study focus on this MFS Mdr transporter, the transport function and mechanism of SotB are systematically studied and analyzed in this work. Initially, the transport activity of SotB to L-arabinose was successfully verified by cell based radiotracer uptake assay. On this basis, the functional roles of substrate binding pocket’s key residues in SotB were further explored by the surface plasmon resonance (SPR) affinity assays, molecular docking analysis, and the L-arabinose inhibition assays. In addition, by analyzing the structure of SotB2 predicted by AlphaFold 2, the key residues responsible for the difference of substrate recognition between SotB and SotB2 was characterized for the first time. Finally, a relatively detailed transport model of SotB based on related results in this study was proposed. Altogether, the novel findings presented in this study positively contribute to the knowledge of MFS Mdr transporters like SotB, especially about the structure and function coupling mechanism.

## Materials and methods

### Strains, plasmids, and growth conditions

*Escherichia coli* MG1655 was used for gene deletion, *E. coli* DH10B was used for gene cloning and *E. coli* C43(DE3) was used for protein expression. All *E. coli* strains were cultivated at 37°C with 220 rpm agitation in LB medium (Becton, Dickinson and Company, America) supplemented with antibiotics, rhamnose and/or sucrose if necessary. All strains, plasmids and primers used in this study are listed in Supplementary Table S1.

### Gene cloning and mutagenesis

The full-length *sotB* and *lapC* genes from the *E. coli* MG1655 genome were subcloned into the cloning vector pET28a, together with the fusion of a C-terminal 8 × His tag. Site-directed variants were produced by the ClonExpress Ultra One Step Cloning Kit (Vazyme Biotechnology Co., Ltd., Nanjing, China) with the plasmid pET28a/*sotB* as the template and verified for fidelity by DNA sequencing in Tsingke Biotechnology Co., Ltd. (Beijing, China).

### Gene deletion of *sotB*

The target *sotB* gene was in-frame deleted by the *in vitro* pEcCas/pEcgRNA system (Li et al., 2021). The related gRNA sequence (5*′*-CGTTAATTTGGTCAATCATT-3*′*) for *sotB* gene knock-out was designed by Benchling online server^2^ with a length of 20 nt. Plasmid pEcgRNA was digested with *Bsa*I to generate linearized pEcgRNA carrying the overhangs 5*′*-TAGT-3*′* and 5*′*-AAAC-3*′*; the linearized pEcgRNA was either used immediately or frozen at −80°C for subsequent use. Then, the Oligonucleotides primer gRNA-F (5*′*-TAGTCGTTAATTTGGTCAATCATT-3*′*) and gRNA-R (5*′*-AAACAATGATTGACCAAATTAACG-3*′*) were synthesized and annealed in a reaction mix consisting of 35 μl ddH_2_O, 5 μl T4 ligase buffer (Vazyme), 5 μl (10 μM) gRNA-F, and 5 μl (10 μM) gRNA-R. The reaction mix was incubated at 95°C for 5 min, and the temperature was then gradually reduced by 5°C–10°C per minute. The reaction was finally held at 16°C for 10 min. The annealed dsDNA was then diluted 200-fold and 1 μl of the diluted DNA was ligated to 1 μl of *Bsa*I-linearized pEcgRNA in a mixture of T4 ligase buffer and T4 ligase for 1 h at 16°C. The ligation product was transformed into DH10B to select for plasmid pEcgRNA/*sotB*. Positive clones were selected on LB plates supplemented with 50 μg/ml spectinomycin.

For genome editing, 100 μl of *E. coli* MG1655::pEcCas competent cells was mixed with 100 ng of pEcgRNA/*sotB* plasmid and 400 ng of donor DNA, and the mixture was electroporated in a precooled 2 mm Gene Pulser cuvette (Bio-Rad, Hercules, United States) at 2.5 kV. The electroporation mixture was immediately suspended in 1 ml of fresh LB medium. Cells were recovered by incubating at 37°C for 1 h before spreading on LB plates containing kanamycin (50 μg/ml) and spectinomycin (50 μg/ml), and the plates were then incubated overnight at 37°C. After electroporation and recovery of transformants on selection plates, individual colonies were randomly picked and verified by colony-PCR and DNA sequencing.

To eliminate the pEcgRNA/*sotB* and pEcCas plasmids, the positive mutants were grown in liquid LB medium containing rhamnose and kanamycin for 6 h. The cells were then transferred to liquid LB medium without any antibiotic and grown further for 2 h. Single colonies were isolated by plating the cells on LB plates containing sucrose. The colonies were then screened on LB plates, LB plates with kanamycin, and LB plates with spectinomycin. Colonies that grew only on LB plates were cured of both pEcgRNA and pEcCas. The pEcCas plasmid was successfully eliminated in this study.

### Expression and purification of LapC, SotB, and SotB related variants

The *lapC*, *sotB*, and *sotB*-related variants genes were cloned into pET28a separately, and the expression constructs were subsequently transformed into *E. coli* C43(DE3). Expression and purification of all proteins were carried out in a similar protocol. The overnight culture was inoculated into 900 ml of LB medium with kanamycin (50 μg/ml), and the cells were grown at 37°C until an OD_600_ of 0.8 and then induced with 0.1 mM isopropyl-β-D-1-thiogalactopyranoside (IPTG) for 20 h at 20°C. After that, the cells were collected, homogenized in the buffer containing 50 mM HEPES (pH 7.5) and 300 mM NaCl, and lysed by homogenization for 35 min on ice. Cell debris was removed by 10,000 g centrifugation for 10 min. The supernatant was collected and ultracentrifuged at 230,000 g for 1 h. Membrane fraction was collected and incubated with 1.5% (w/v) dodecyl-b-D-maltopyranoside (DDM, Anatrace) for 1 h at 4°C. After another centrifugation step at 20,000 g for 1 h, the supernatant was collected and loaded on Ni^2+^-nitrilotriacetate affinity resin (Ni-NTA, Cytiva) and washed with 50 mM HEPES (pH 7.5), 300 mM NaCl, 40 mM imidazole and 0.02% DDM. The protein was eluted from the affinity resin with 50 mM HEPES (pH 7.5), 300 mM NaCl, 300 mM imidazole and 0.02% DDM and concentrated to 1 ml before further purification by gel filtration (Superdex 200 Increase 10/300 Gl, Cytiva). The buffer for gel filtration contained 20 mM HEPES (pH 7.5), 100 mM NaCl and 0.02% DDM. The peak fraction was collected.

### Cell based uptake assay

The cell-based uptake assay was performed with the following protocol. Plasmids pET28a and pET28a/*sotB* were transformed into *E. coli* C43(DE3) competent cells, respectively. Then, these two kinds of cells were grown in LB medium at 37°C and induced with 0.2 mM IPTG for 1 h, when the cell density reached an OD_600_ of about 1.5. Cells were then harvested by centrifugation. After being washed three times with MK buffer (150 mM KCl, 5 mM MES, pH 6.5), the cells were resuspended in the same buffer to an OD_600_ of 2.0. To measure the transport activity of SotB protein, took 5 ml of the above cell suspension, respectively, and added ^3^H-L-arabinose (American Radiolabeled Chemicals) at a final concentration of 0.1 μM. All the reactions lasted 1 h and were stopped by diluting the cell suspension with 5 ml ice-cold MK buffer. Cells were then harvested by centrifugation. After being washed three times with ddH_2_O, the cells were resuspended in 5 ml ddH_2_O, respectively. Then the cells were added to the sample bottle, respectively, and mixed with the same volume of excess scintillation, and then taken for liquid scintillation counting. All the experiments were repeated at least three times.

### Surface plasmon resonance affinity assay of SotB and its variants

The instrument used in surface plasmon resonance (SPR) affinity assays was OpenSPR (Nicoya, Canada) and the chip used was COOH sensor chip (Nicoya, Canada). SotB and its variants were as ligands. IPTG, L-arabinose, D-xylose, D-glucose, D-fructose, and D-mannose were as small molecules. Using HSB-EP buffer (10 mM HEPES, 150 mM NaCl, 3 mM EDTA, 0.005% Tween-20, pH7.4) as test buffer and setting flow rate 150 μl/min after installed COOH chip according to OpenSPRTM instrument standard operating procedure. After the signal baseline was reached, loading 200 μl 80% isopropyl alcohol and ran for 10 s to drain bubbles. After reaching baseline, the sample ring was rinsed with HSB-EP buffer and emptied with air. After the signal reached the baseline, loading 200 μl EDC (400 mM, Sigma)/NHS (100 mM, Sigma; 1:1) solution with a flow rate 20 μl/min for 4 min. Then, loading 200 μl ligand (SotB/SotB variants) diluted by the activation buffer (sodium acetate, 10 mM, pH3.5) and ran for 4 min (20 μl/min). Then the sample ring was rinsed with HSB-EP buffer and emptied with air. Loading 200 μl 1 M ethanolamine (Sigma) blocking solution (20 μl/min, 4 min), rinsed the sample ring with HSB-EP buffer, and emptied the sample ring with air. Observing baseline for 5 min to ensure stability. The small molecules were diluted with PBS buffer (2 mM of KH_2_PO_4_, 10 mM of Na_2_HPO_4_, 2.7 mM of KCl, and 137 mM of NaCl at pH 7.4), and the concentration was shown in the experimental results. The flow rate for small molecules was performed at 20 μl/min. The binding time of small molecule and ligand was 240 s, and the dissociation was 240 s. The analysis software used for the experimental results was TraceDrawer, and the analysis method was one-to-one analysis model.

### Assessment of the effect of L-arabinose on *Escherichia coli* MG1655 growth curve

In the process of *sotB* gene knockout, two plasmids were introduced into *E. coli* MG1655 strain: the plasmid pEcCas expressing Cas9 protein (kanamycin resistance) and the plasmid pEcgRNA/*sotB* expressing related gRNA (spectinomycin resistance). The pEcCas plasmid was successfully eliminated in this study. Considering that the existence of plasmid pEcgRNA/*sotB* does not affect the L-arabinose inhibition assay, the plasmid pEcgRNA/*sotB* was not eliminated in this study. Thus, the *sotB*-deletion strain has resistance to spectinomycin. In order to carry out the related L-arabinose inhibition assay more strictly, the same plasmid (pEcgRNA/*sotB*) was then transformed into intact *E. coli* MG1655 cells, and the growth conditions of the two strains were measured as follows: The *E. coli* MG1655 cells (intact cells and *sotB-*deletion cells) were grown in liquid LB medium containing spectinomycin (50 μg/ml) at 37°C for overnight. The cells were then diluted to an OD_600_ value of 0.8 with liquid LB medium containing spectinomycin (50 μg/ml). Then, the cells were further diluted 1,000 times with liquid LB medium containing spectinomycin (50 μg/ml). The diluted cells were added to a 96-well plate (200 μl/well). Then, L-arabinose was added with final concentrations of 0 μM, 10 μM, 100 μM, 1 mM, 10 mM, and 100 mM, respectively. The 96-well plate was incubated at 37°C, and the OD_600_ value was measured every 1 h with a microplate reader. For the competition assay, the intact MG1655 cells diluted 1,000 times were divided into two parts and then added into 96-well plate (200 μl/well). One part was directly added with D-xylose at a final concentration of 0, 1, and 10 mM, respectively; the other part was first added with L-arabinose at a final concentration of 10 mM, then D-xylose was added with final concentrations of 0, 1, and 10 mM, respectively. The method of growth curve measurement is the same as above. All the experiments were repeated at least three times.

### Assessment of the effect of L-arabinose on *Escherichia coli* C43 growth curve

The *E. coli* C43 cells containing wild-type SotB or a given SotB mutant (E29A, H115A, W119A, G153A, G153E, G157E, L337A, S339A, and N343A) were grown in 100 ml LB medium containing kanamycin (50 μg/ml) at 37°C for overnight. The cells were then diluted to an OD_600_ of 1 with liquid LB medium containing kanamycin (50 μg/ml), and induced with 0.2 mM IPTG for 1 h. The cells were then diluted to an OD_600_ of 0.8 with liquid LB medium containing kanamycin (50 μg/ml). The cells were further diluted 1,000 times with liquid LB medium containing kanamycin (50 μg/ml), and IPTG with a final concentration of 0.4 mM was added. The diluted cells were added to a 96-well plate (200 μl/well). Then, L-arabinose was added with a final concentration of 10 mM. The 96-well plate was incubated at 37°C, and the OD_600_ was measured every 1 h with a microplate reader. Cells transformed with the lipopolysaccharide signal transducer LapC in pET28a vector were used as control (ctrl). All the experiments were repeated at least three times.

### Multiple sequence alignment and protein structure prediction by AlphaFold2

The sequences of SotB and other related proteins in this study were uploaded to ESPript 3.0 server^3^ to build the multiple sequence alignment with the default settings (Robert and Gouet, 2014). AlphaFold 2^4^ was applied to predict the structure of SotB2 with high quality following its official protocol (Mirdita et al., 2022). The predicted structure was submitted to the UCLA−DOE LAB-SAVES v6.0 web server^5^ for quality assessment. All molecular structures were visualized using the PyMOL software.^6^

### Docking of IPTG, L-arabinose, D-xylose, and melibiose

The input files of proteins (SotB and SotB2) and small molecules (IPTG, L-arabinose, D-xylose, and melibiose) were prepared with AutoDock Tools, with reference to the X-ray crystal structures (PDB ID: 6KKI, 2ARC, 3XIS, and 4AL9) containing the related small molecules. The prepared small molecules were docked into the active site of the chosen structure using Autodock Vina. A box of size 30 × 30 × 30 Å centered on the selected active site residues (the active site was defined as the region where the IPTG was bound in the co-crystal structure of the SotB—IPTG complex) was used to restrict the search space of each docked small molecule. The docking protocol was first validated by redocking the IPTG into the active site of SotB that resulted in the docked pose of IPTG to be very similar to the experimentally observed position and conformation of IPTG. Docking was carried out using rigid SotB/SotB2 proteins and flexible small molecules. The top scoring binding pose was selected for further analysis. All molecular structures were visualized using the PyMOL software.^7^

## Results

### Sequence analysis of SotB with structure-solved MFS Mdr transporters

**Figure 1:**
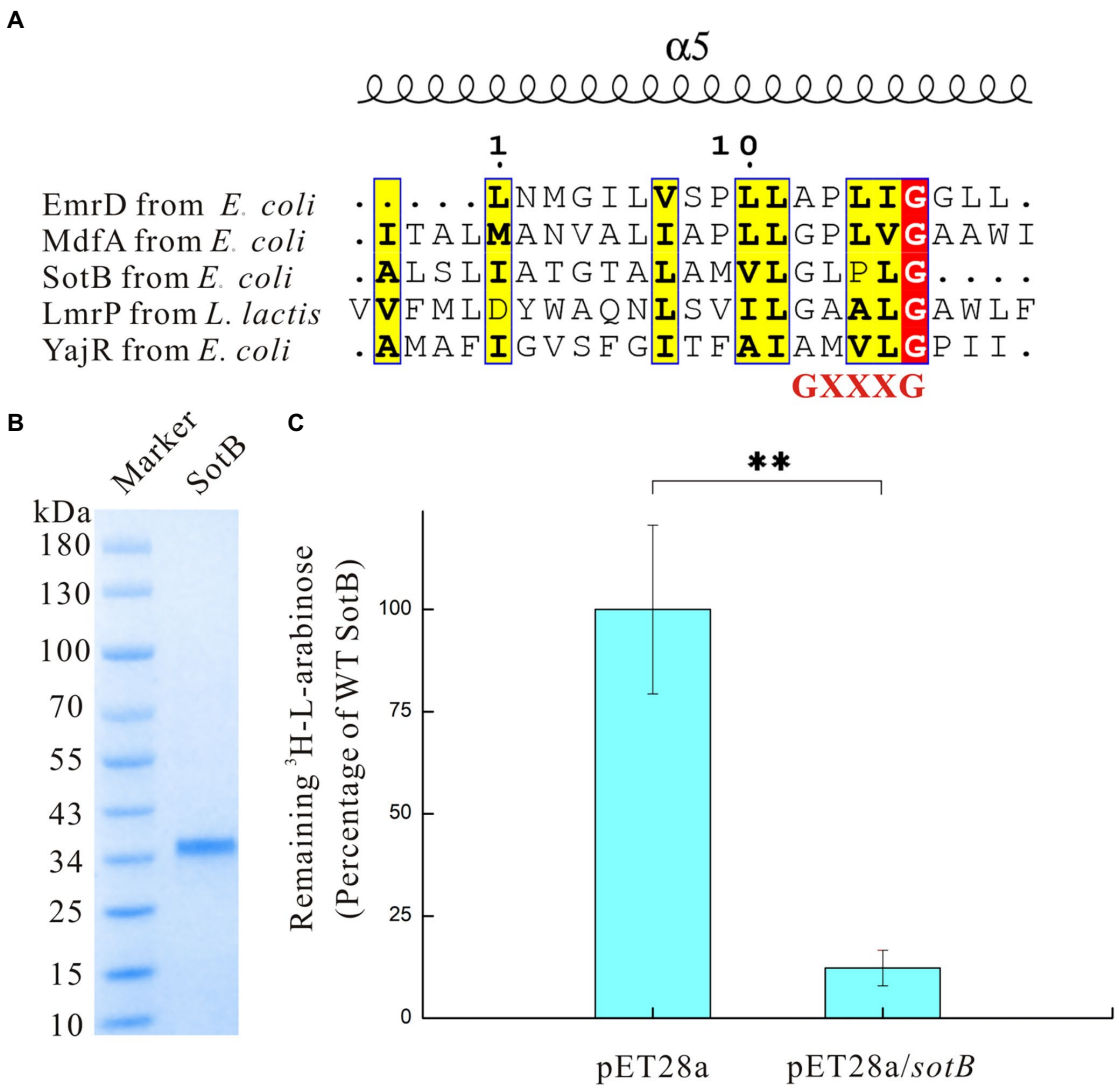
Sequence analysis and functional characterization of SotB in *Escherichia coli*. **(A)** Analysis of Motif C site on TM5 of SotB, EmrD, MdfA, LmrP, and YajR. Motif C site is a conservative site contained in the antiporter. Sequence alignment results in this study show that among the five antiporters whose structures have been resolved, the protein sequence of the conservative Motif C site is GXXXG; **(B)** SDS-PAGE analysis of SotB (MW: 42.5 kDa). Due to the hydrophobicity of membrane proteins, the position of the membrane protein band will be 10–20 kDa lower than the position corresponding to the theoretical molecular weight during SDS-PAGE analysis; **(C)** Activity of the recombinant SotB. Transport activity of the recombinant wild-type SotB was verified using a cell-based ^3^H-L-arabinose uptake assay. The empty vector pET28a was used as a negative control, ^**^p < 0.01.

As a major facilitator superfamily multidrug (MFS Mdr) transporter, SotB from *Escherichia coli* also belongs to the DHA12 family. Besides SotB, there are currently only four structures available from MFS Mdr transporters EmrD (TCDB ID: 2.A.1.2.9), YajR (TCDB ID: 2.A.1.2.60), MdfA (TCDB ID: 2.A.1.2.19), and LmrP (TCDB ID: 2.A.1.2.5). These four structure-solved MFS Mdr transporters were used to create the protein alignment with SotB for the residue conservation analysis. Previous studies have shown that antiporters including MFS Mdr transporters usually have the conserved Motif C site on TM5 and this conserved Motif C site is expected to have an essential role in antiporter activity (Jesus et al., 2005; Yaffe et al., 2013). However, different studies have different definitions of Motif C site. Ginn et al. defined Motif C site as gXXGPxiGGxl (Ginn et al., 2000), whereas Heng et al. defined Motif C site as PLXGPXXG (Heng et al., 2015). The common sequence defined by the two studies is GXXXG. According to our sequence analysis, among the five MFS Mdr transporters whose structures were solved, the most conserved site on TM 5 is GXXXG (Figure 1A). Therefore, it can be confirmed that the GXXXG sequence should be the most typical conserved Motif C sequence of the antiporters. The overall sequence similarities between these five transporters are very low, ranging from 13.4% to 20.2%, and SotB has the highest sequence similarity with LmrP derived from *L. lactis* at 16.4% (Supplementary Table S2). This result is consistent with the characteristic that most MFS members share low sequence conservation of 12%–18% (Madej et al., 2014). The sequence alignment results also showed that two aliphatic residues (G157 and L377) and one alkaline residue (R78) are fully conserved in SotB (Supplementary Figure S1). Among these residues, G157 is the most conserved amino acid in Motif C on TM5, L377 is located on TM12, while R78 is located on the loop region connecting TM2 and TM3 (Supplementary Figure S2).

Like SotB, MdfA derived from *E. coli* also belongs to the MFS Mdr transporter, which can transport a variety of substances (Edgar and Bibi, 1997; Fluman et al., 2012). The sequence similarity between SotB and MdfA is 13.4% (Supplementary Table S2). MdfA Q131 is located in the cytoplasmic region, studies have shown that a single mutation Q131R in MdfA has made it possible to obtain highly ordered crystals that diffracted X-ray down to a resolution of 2 Å (Heng et al., 2015). Replacing Q131 by an arginine partially disrupts the charge asymmetry and thus might lead to electrostatic repulsion between the two halves of the rim and possibly stabilization of MdfA Q131R in a specific, partially inward-open conformation (Heng et al., 2015), and this result showed that some residues far from the substrate binding site can be just as important for proteins to function properly as those residues directly coordinating the substrate. Alignment between the structures of SotB (PDB ID: 6KKI) and MdfA (PDB ID: 4ZP0) exhibited an r.m.s.d. (root mean squared deviation) of 2.8 Å over 359 residues calculated by DALI server (Holm, 2020). Interestingly, in SotB, the same position as MdfA Q131 is R128 (Supplementary Figure S2). Therefore, SotB R128 may have the same function as MdfA Q131R, that is, it may stabilize the conformation of SotB to some extent and this may explain why relatively high-quality crystals of wild-type SotB have been successfully obtained by ordinary crystallography method (Xiao et al., 2021).

### L-arabinose transport activity of SotB in *Escherichia coli*

Previous studies have demonstrated that SotB can mediate the transport of L-arabinose by using relevant genetic experiments (Bost et al., 1999; Carole et al., 1999; Koita and Rao, 2012). Due to the low promoter activity, the expression of wild-type SotB in *E. coli* is very low and almost silent (Bost et al., 1999), therefore, comparing the uptake effect of the wild-type strain and the *sotB* overexpression strain by using radiolabeled substrate can be regarded as a good way to explore the transport function of SotB.

**Figure 2:**
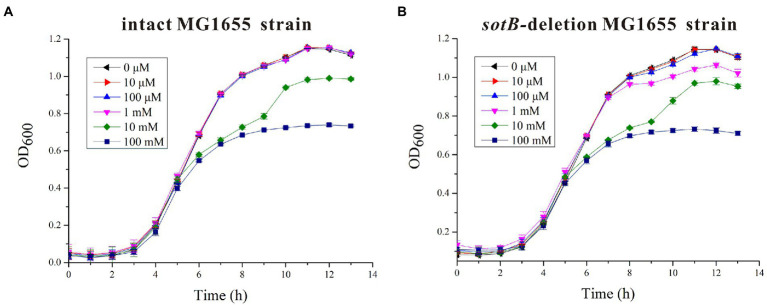
Time-dependent *Escherichia coli* MG1655 growth assays in the presence of different concentrations of L-arabinose. **(A)** The growth curves of intact *E. coli* MG1655 strain under a serial concentrations of L-arabinose; **(B)** The growth curves of *sotB*-deletion *E. coli* MG1655 strain under a serial concentrations of L-arabinose.

The *sotB* gene from *E. coli* was cloned and expressed. The recombinant SotB protein was purified with N-Dodecyl-β-D-Maltopyranoside (DDM) by Ni^2+^ affinity and size-exclusion chromatography. (Figure 1B; Supplementary Figure S4). To characterize the transport ability of SotB on L-arabinose, the radiotracer based uptake assay was carried out. Expressed in the *E. coli* wild-type SotB confers significant reduced accumulation of ^3^H-L-arabinose relative to empty vector pET28a (Figure 1C), and this result indicated that the expressed SotB protein transported part of ^3^H-L-arabinose from the intracellular to extracellular. As far as we know, this is the first time that the efflux function of SotB on L-arabinose is measured directly by overexpression of *sotB* gene combined with a radiotracer uptake assay. The *sotB* gene in *E. coli* MG1655 was successfully knocked out in this study and the growth curves of the intact MG1655 strain and the *sotB*-deletion MG1655 strain were further measured under a serial concentrations of L-arabinose (Supplementary Figure S3A). L-arabinose with final concentrations of 10 mM and 100 mM could significantly inhibit the growth of both intact and *sotB*-deletion MG1655 (Figures 2A,B), indicating that excessive accumulation of L-arabinose was harmful to *E. coli* MG1655. Notably, in the presence of 1 mM L-arabinose, the intact MG1655 grew better compared to *sotB*-deletion MG1655 (Supplementary Figure S3B). These results suggest that excessive L-arabinose can prohibit the growth of MG1655, and deletion of *sotB* gene will prevent the efflux of L-arabinose making the strain more sensitive to L-arabinose.

### Functional analysis of key amino acids in SotB transport pocket

**Figure 3:**
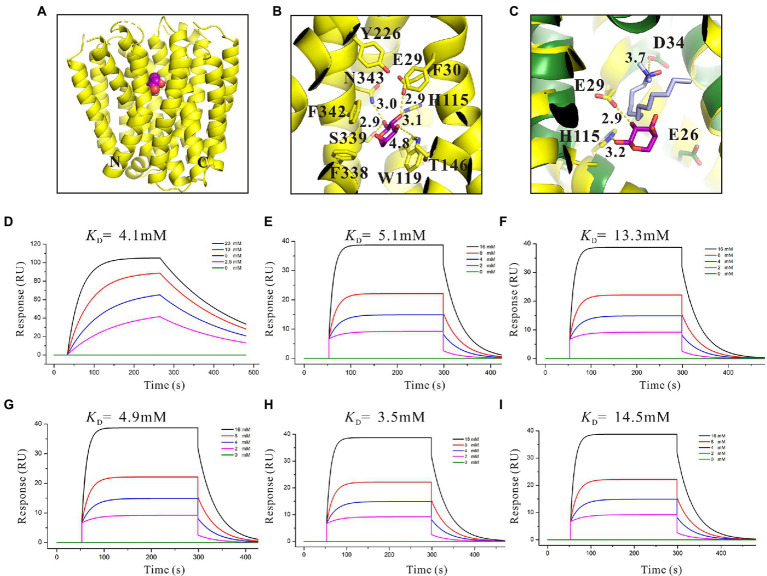
Structural basis and binding affinity analysis of SotB/SotB variants with its substrate L-arabinose. **(A)** The overall structure of docked SotB-L-arabinose complex. The SotB structure is shown as yellow cartoon, and the L-arabinose molecule is shown as purple sphere; **(B)** Coordination of L-arabinose. Hydrogen bonds are represented with yellow dashed lines, L-arabinose is shown as purple sticks; **(C)** Comparison of the protonation site of SotB and MdfA. The MdfA structure is shown as dark green cartoon. The substrate LDAO of MdfA is represented as gray sticks; **(D)** Binding affinity of L-arabinose with SotB; **(E)** Binding affinity of L-arabinose with E29A; **(F)** Binding affinity of L-arabinose with H115A; **(G)** Binding affinity of L-arabinose with W119A; **(H)** Binding affinity of L-arabinose with S339A; **(I)** Binding affinity of L-arabinose with N343A. The colored lines represent the concentrations of L-arabinose used in SPR assays.

The three-dimensional (3D) structures of SotB from *E. coli* were determined by Xiao *et al* in multiple inward-open states and an occluded conformation with substrate IPTG bound, however, the complex structure of SotB-L-arabinose has not been solved until now (Xiao et al., 2021). The substrate L-arabinose was docked into the structure of SotB (PDB ID: 6KKI) for analysis in this study (Figure 3A). The structure-solved SotB was used as the receptor, removed IPTG from its active pocket, and used L-arabinose molecule for docking. With the position of IPTG in the complex structure as the center, the size of the docking box was set as 30 × 30 × 30. According to our docking model, the L-arabinose molecule is surrounded by both polar and aromatic residues, including E29, F30 on TM1; H115, W119 on TM4; T146 on TM5; Y226 on TM7, and F338, S339, F342, N343 on TM11 (Figure 3B). Among these residues, E29/H115, W119, and S339/N343 can interact with C2-OH, C3-OH, and C1-OH of L-arabinose *via* hydrogen bond, respectively (Figure 3B). These hydrogen bonds can stabilize the L-arabinose in the transport center. In addition, two aromatic residues W119 and F342 stack together to form a limited site for L-arabinose (Figure 3B).

The binding of substrate to protein is one of the most basic and important processes. Understanding the binding affinity between SotB and L-arabinose can better explain the mechanism of SotB-mediated L-arabinose transport. Thus, the binding affinity of them was measured by surface plasmon resonance (SPR) technique, and the result showed that SotB has an affinity with L-arabinose, with a *K*_D_ (equilibrium dissociation constant) value of 4.1 mM (Figure 3D). Among several reported MFS transporters whose binding affinities with substrates have been measured, the binding affinities between FucP and the substrate fucose, MdfA and the substrate Chloramphenicol (Cm), MelB and the substrate melibiose is 0.47, 0.075, and 1 mM, respectively (Dang et al., 2010; Ethayathulla et al., 2014; Heng et al., 2015). These results show that SotB, FucP, MdfA, and MelB have low binding affinities with their substrates, representing the substrate binding affinity feature of MFS transporter family.

**Figure 4:**
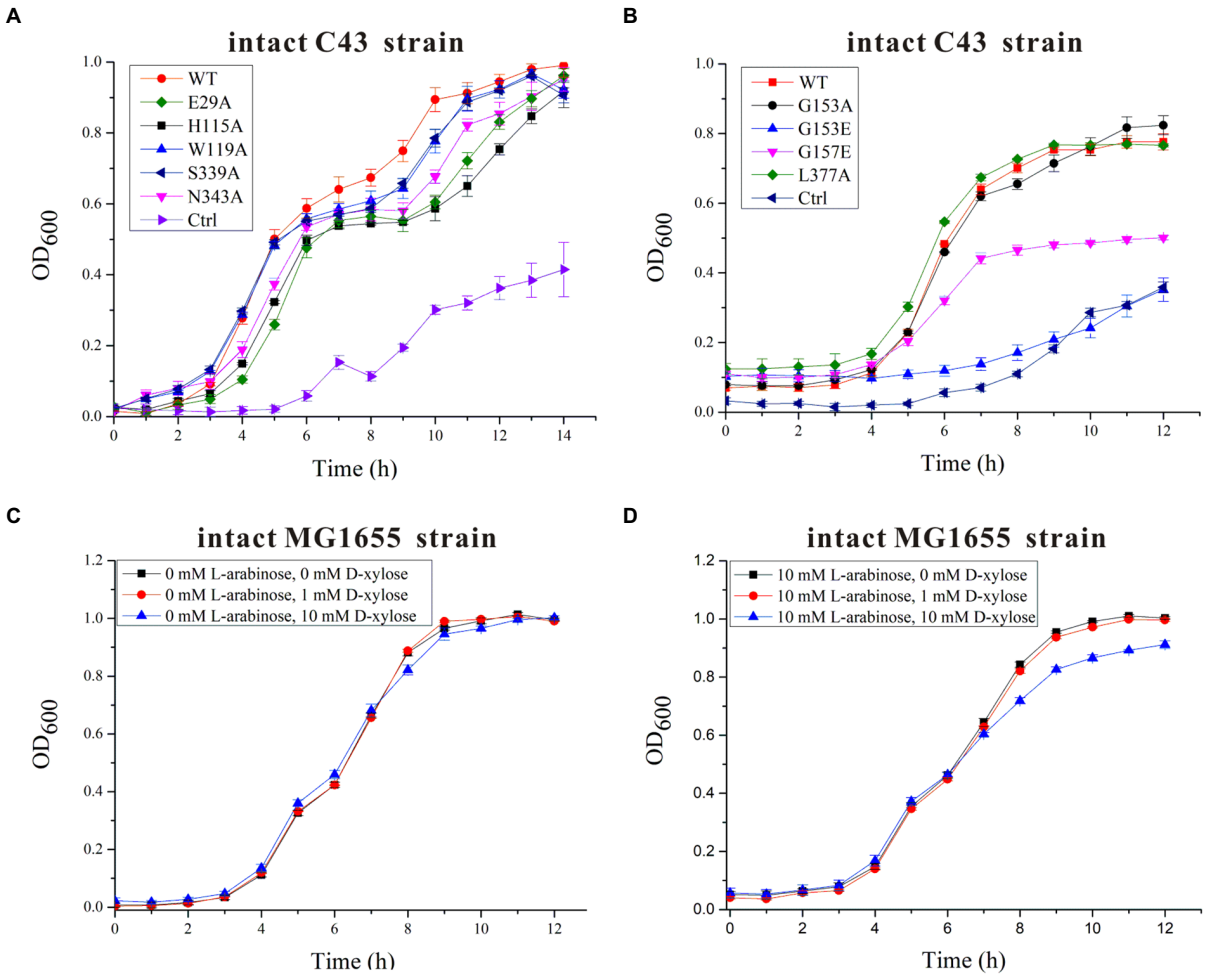
Time-dependent *Escherichia coli* C43 and MG1655 growth assays in the presence of 10 mM L-arabinose and/or different concentrations of D-xylose. **(A)** The growth curves of intact *E. coli* C43 strain containing wild-type (WT) SotB or a given SotB mutant (E29A, H115A, W119A, S339A, and N343A) were measured in the presence of 10 mM L-arabinose. Cells transformed with the lipopolysaccharide signal transducer LapC in pET28a vector were used as control (ctrl); **(B)** The growth curves of intact *E. coli* C43 strain containing wild-type (WT) SotB or a given SotB mutant (G153A, G153E, G157E, and L377A) were measured in the presence of 10 mM L-arabinose. Cells transformed with the lipopolysaccharide signal transducer LapC in pET28a vector were used as control (ctrl); **(C)** The growth curves of the intact *E. coli* MG1655 strain were measured in the presence of different concentrations (0, 1, and 10 mM) of D-xylose; **(D)** The growth curves of the intact *E. coli* MG1655 strain were measured in the presence of 10 mM L-arabinose and different concentrations (0, 1, and 10 mM) of D-xylose. Data represented in graphs were collected from three biological replicates and presented as means ± standard deviations.

On the basis of residue locations, hydrogen bonding reactions and possible polar contacts between residues, five amino acids (E29, H115, W119, S339, and N343) were supposed to play important roles in the function of SotB. These five mutants were expressed respectively, and the monoalanine mutation at these five sites did not affect the expression of the target protein (Supplementary Figure S5). SPR results showed that variants H115A and N343A displayed reduced binding affinities toward L-arabinose, 3.2- and 3.5-fold lower than the wild-type protein respectively, whereas E29A, W119A, and S339A maintained a comparable L-arabinose binding affinity with the wild-type protein (Figures 3E–I). According to the SPR results, the binding affinities of W119A and S339A with L-arabinose were almost unchanged compared with that of wild-type SotB (Figures 3E,G,H), which indicated that alanine mutation of W119 and S339 did not affect the binding of the L-arabinose to SotB. The L-arabinose inhibition assay results showed that compared with wild-type strain, alanine mutations at W119, S339, and N343 all inhibited the growth of the bacteria with varying degrees (Figure 4A). Considering that our radiotracer uptake assay has displayed the expelling ability of SotB, these mutants should most likely affect the L-arabinose expelling activity of SotB, and thus lead to the growth defect by the accumulation of L-arabinose in the bacteria. The above SPR and biochemical results suggest that W119 and S339 may be involved in the L-arabinose efflux process, whereas N343 may play an important role in L-arabinose recognition process.

The presence of an acidic residue in TM1, TM4, TM7, or TM10 of proton-coupled sugar porters is key for H^+^ coupling (Yan, 2013). In the transport pocket of SotB, the most possible residues that can undergo cycles of protonation and deprotonation along the transport path are E29 on TM1 and H115 onTM4 (Figure 3C). The roles of the two residues were also manifested by the L-arabinose inhibition assays. Replacement of H115 by Ala, resulted in significant decrease of SotB-mediated active transport of L-arabinose (Figure 4A). Combined with the SPR result, the mutation from H115 to A115 significantly reduced the binding affinity of SotB to L-arabinose (Figures 3D,F), these observations suggest that H115 is involved in the substrate recognition process. In MdfA, the highly conserved TM1 acidic residue D34 is required to achieve H^+^-coupled efflux of substrate. The positively charged N1 of LDAO is 3.7 Å away from the side-chain carboxylate of D34, likely forming a long-range charge–charge interaction (Figure 3C). The side chain carboxylate group of E29 is 2.9 Å away from the C2-OH of L-arabinose (Figure 3C), which is similar with the distance between MdfA D34 and N1 of LDAO (Figure 3C), and the binding affinity of E29A with L-arabinose is almost identical compared to that of the wild-type SotB with L-arabinose (Figures 3D,E), indicating that it does not participate in the substrate recognition process. Moreover, replacement of E29 by Ala also resulted in significant decrease of SotB-mediated active transport of L-arabinose (Figure 4A), so E29 is likely to be responsible for the protonation process. Taken together, our experimental results indicate that E29, H115, W119, S339, and N343 are all very important for the active transport activity of SotB.

### Binding affinity analysis of IPTG with SotB/SotB variants

**Figure 5:**
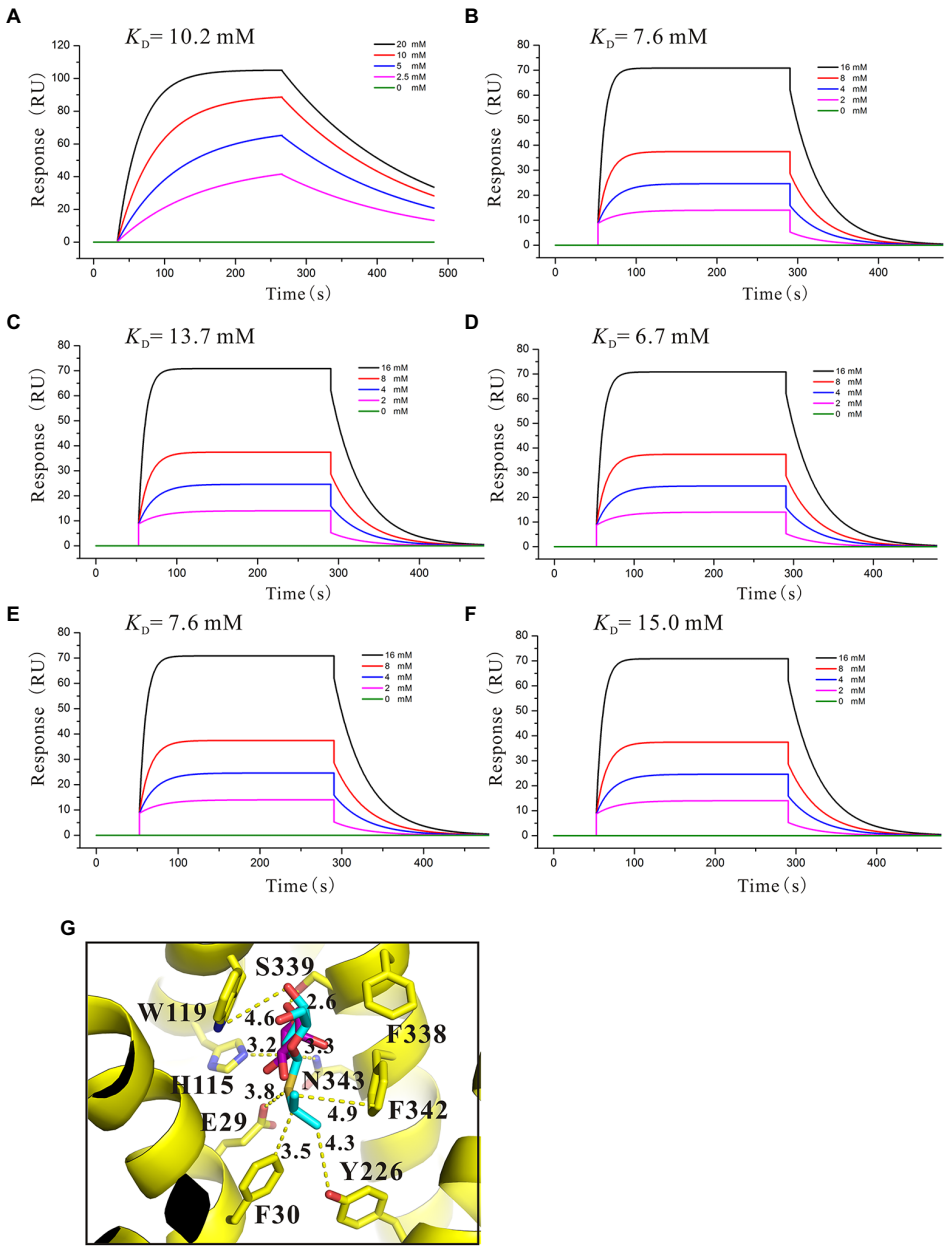
Binding affinity analysis of IPTG with SotB/SotB variants. **(A)** Binding affinity of IPTG with SotB; **(B)** Binding affinity of IPTG with E29A; **(C)** Binding affinity of IPTG with H115A; **(D)** Binding affinity of IPTG with W119A; **(E)** Binding affinity of IPTG with S339A; **(F)** Binding affinity of IPTG with N343A. The colored lines represent the concentrations of IPTG used in SPR assays; **(G)** Coordination of IPTG by SotB. Hydrogen bonds are represented with yellow dashed lines. IPTG and docked L-arabinose are represented with cyan and purple sticks, respectively.

By using relevant genetic methods, Carole et al. revealed that SotB may also transport IPTG (Carole et al., 1999). Notably, IPTG was used to induce the expression of SotB protein in this study. The purification result of SotB protein (Figure 1B) and the successful measurement of its transport function to L-arabinose (Figure 1C) indicate that although SotB may expel IPTG, IPTG can still induce the normal expression of SotB protein. Xiao *et al* recently solved the structure of SotB-IPTG complex with a resolution of 3.1 Å (Xiao et al., 2021), and the structural information showed that E29, H115, W119, S339, and N343 can form hydrogen bonds with IPTG (Figure 5G). Different from L-arabinose, the hydrophobic tail of IPTG can make contacts with the side chains of F30, Y226, and F342 through van der Waals interactions (Figure 5G). These van der Waals interactions may make IPTG more stable in the active cavity of SotB. The binding affinity between SotB and IPTG showed that IPTG bind to SotB with a *K*_D_ value of 10.2 mM, which is nearly 2.5-fold lower to the binding affinity between L-arabinose with SotB (Figures 3D, 5A). Interestingly, the binding affinities between SotB variants (E29A, H115A, W119A, S339A, N343A) and IPTG did not change significantly compared with the binding affinity between wild-type SotB and IPTG (Figures 5B–F). However, there is no direct experimental evidence proving that SotB can transport IPTG directly so far. Therefore, whether SotB can transport IPTG directly remains to be verified by relevant experiments.

### Binding affinity analysis of L-arabinose analogs with SotB

**Figure 6:**
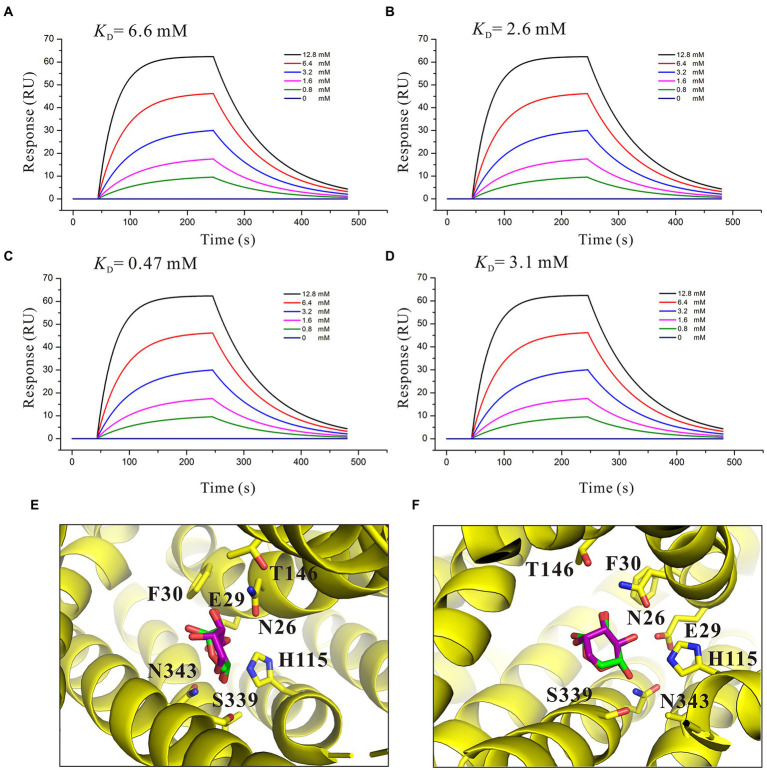
Affinity analysis of SotB with L-arabinose analogs. **(A)** Binding affinity of D-fructose with SotB; **(B)** Binding affinity of D-glucose with SotB; **(C)** Binding affinity of D-xylose with SotB; **(D)** Binding affinity of D-mannose with SotB. The colored lines represent the concentrations of corresponding small molecules; **(E)** Comparison of the conformations and positions of the docked L-arabinose and D-xylose. The L-arabinose and D-xylose molecules are shown as purple and green sticks, respectively; **(F)** Another view that is different from E for comparison of the conformations and positions of the docked L-arabinose and D-xylose.

To better understand the characteristic of binding affinity between SotB and its substrate, the binding affinities of a series of L-arabinose analogs with SotB were measured. SPR results showed that the binding affinities of D-fructose (*K*_D_ = 6.6 mM), D-glucose (*K*_D_ = 2.6 mM) and D-mannose (*K*_D_ = 3.1 mM) with SotB are similar with the binding affinity between L-arabinose (*K*_D_ = 4.1 mM) and SotB (Figures 3D, 6A,B,D). So far, no studies have shown that SotB can transport D-fructose, D-glucose, and D-mannose. Unexpectedly, the binding affinity of D-xylose to SotB is 0.47 mM, which is 8.7-fold higher than that of L-arabinose to SotB (Figures 3D, 6C). From our docking result, the positions and conformations of L-arabinose and D-xylose are almost identical (Figures 6E,F). The binding affinity between XlyE and its substrate D-xylose is 0.35 mM (Sun et al., 2012), which is almost the same as the binding affinity between SotB and D-xylose, however, the previous study has shown that SotB cannot transport D-xylose (Koita and Rao, 2012). Altogether, our data suggested that SotB has high transport specificity for L-arabinose. At the same time, the millimolar level binding affinity also makes the transport process faster, and improves the transport efficiency. The above results also indicate that sugars even with very similar structures, such as D-xylose, cannot be transported by SotB. D-xylose has much higher binding affinity with SotB compared with L-arabinose, and this may make D-xylose competing with L-arabinose when binds to SotB, and exist as an inhibitor of L-arabinose transport process. To test this possibility, firstly, D-xylose was added to intact MG1655 strain with final concentrations of 0, 1, and 10 mM, respectively, without the addition of L-arabinose. The result showed that the addition of 1 and 10 mM D-xylose did not cause any growth inhibition (Figure 4C). Then, in the presence of 10 mM L-arabinose, the same final concentrations (0, 1, and 10 mM) of D-xylose were added to the intact MG1655 strain, respectively, and the growth conditions were measured. The result showed that in the presence of 10 mM L-arabinose, 10 mM D-xylose will inhibit the growth of MG1655 (Figure 4D). This suggest that excessive D-xylose can compete with L-arabinose, during the L-arabinose expelling by SotB, and eventually leading to the growth inhibition. This result confirms our hypothesis based on our SPR results that D-xylose may be an inhibitor of SotB-mediated L-arabinose transport.

### Structural basis of functional differences between SotB and SotB2

**Figure 7:**
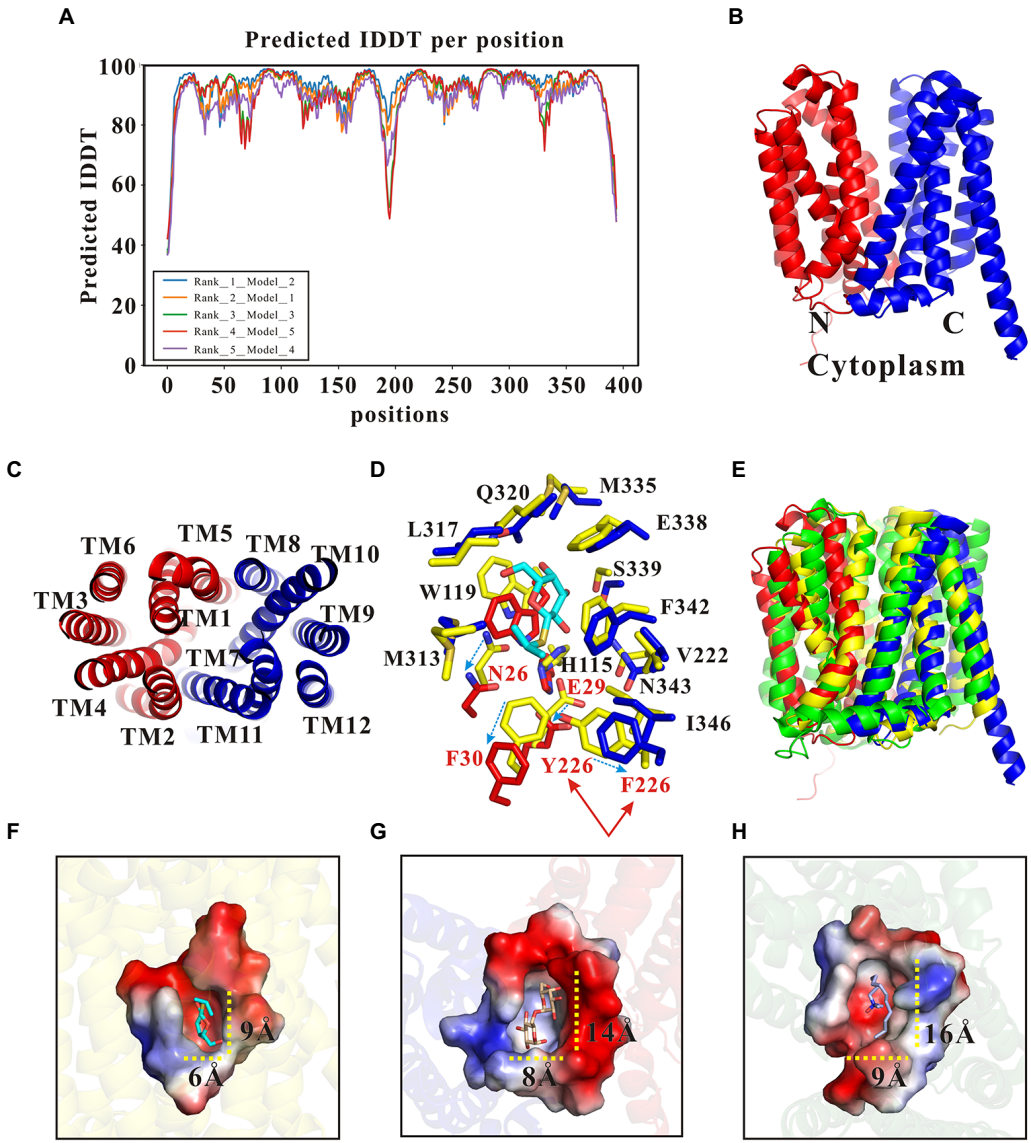
Structural basis of functional differences between SotB and SotB2. **(A)** Five SotB2 structures were generated by the Alphafold 2 with a pIDDT 92.7% (rank_1_model_2), 91.3% (rank_2_model_1), 91.1% (rank_3_model_3), 90.7% (rank_4_model_5), and 88.6% (rank_5_model_4), respectively. The relationship between the reliability of the predicted structure and pIDDT: Very high (pIDDT > 90), Confident (90 > pIDDT > 70), Low (70 > pIDDT > 50), Very low (pIDDT < 50); **(B)** The overall structure of SotB2 (rank_1_model_2) predicted by AlphaFold 2. The N-terminal domain of SotB is shown as red cartoon and the C-terminal domain is shown as blue cartoon; **(C)** Top view of SotB2. TM segments are numbered; **(D)** The superimposition of the transport cavity of SotB and SotB2. The IPTG molecule in SotB structure is shown as cyan sticks. Amino acids from SotB are shown as yellow sticks. Amino acids from the N-terminal domain of SotB2 are shown as red sticks. Amino acids from the C-terminal domain of SotB2 are shown as blue sticks; **(E)** The superimposition of the overall structure of SotB (PDB ID: 6KKI), SotB2 (rank_1_model_2) and MdfA (PDB ID: 4ZP0); **(F)** The surface representation of transport cavity of SotB. The substrate IPTG molecule in SotB is shown as cyan sticks; **(G)** The surface representation of transport cavity of SotB2. The docked substrate melibiose molecule in SotB2 is shown as wheat color sticks; **(H)** The surface representation of transport cavity of MdfA. The substrate LDAO molecule in MdfA is shown as gray sticks. The 3D structures used in **(F–H)** are all belonged to the inward-occluded conformation.

SotB derived from the *Erwinia chrysanthemi* (hereafter named SotB2) has 62.8% protein sequence similarity with SotB derived from *E. coli* (Supplementary Figure S6), however, the transport substrates of the two transporters are quite different. Unlike SotB, SotB2 can transport lactose and melibiose, both of which are disaccharides (Supplementary Figure S7; Condemine, 2000). SotB cannot transport disaccharides despite high protein sequence similarity between SotB and SotB2. Thus, we want to explain this functional difference by analyzing the structural information of the two MFS Mdr transporters. The experimental structure of SotB2 has not been yet revealed. The AlphaFold 2 program has raised a great and well deserved enthusiasm in the biology community due to the overall high accuracy of the 3D protein structures they predicted (Cramer, 2021; Jumper et al., 2021; Pakhrin et al., 2021), thus the SotB2 structure was modeled by AlphaFold 2 in this study. Five structures were generated by AlphaFold 2, which had different predicted local distance difference test (plDDT) scores (Figure 7A). plDDT is a measure of local accuracy of the structure, and the regions with plDDT values larger than 90 are predicted to be highly accurate. Among the five structures, the positions with low reliability (pIDDT < 70) are mainly located in the region of the first and last 20 amino acids, and the loop region of the center connecting the N-terminal and C-terminal (Figure 7A). The rank_1_model_2 showed the highest plDDT score 92.7% among these five structures, which was selected for further analysis (Figure 7A). The SotB2 structure (rank_1_model_2) produced by AlphaFold 2 is in the inward-occluded conformation, which contains the typical MFS fold of two six-helix bundles (N and C domains) with a central cavity, each 6-TMs bundle is itself made up of two 3-TMs segments related by a 180°rotation running parallel to the plane of the membrane (Figures 7B,C). MFS Mdr transporter MdfA exhibits an extremely broad spectrum of drug recognition (Edgar and Bibi, 1997; Fluman et al., 2012). The superimposition of the whole structure of SotB, SotB2, and MdfA revealed that the three structures are very similar overall (Figure 7E), the r.m.s.d. between SotB and SotB2, SotB and MdfA, SotB2 and MdfA is 3.3 Å over 363 residues, 2.8 Å over 359 residues and 3.6 Å over 345 residues, respectively, and the most significant differences were observed in the flexible loops and the SotB2 has a longer TM12 in the C-terminal domain compared with SotB and MdfA (Figure 7E). Surprisingly, the superimposition of the transport pocket of SotB and SotB2 revealed clear conformational differences between them. In SotB, there are a total of 16 amino acids within 6 Å of IPTG, including N26, E29, F30, H115, W119, V222, F226, M313, L317, Q320, M335, E338, S339, F342, N343, and I346. The amino acid composition of the transport pockets of SotB2 and SotB is almost identical according to the results of their superimposition, and the only different amino acid within 6 Å of IPTG is located at position 226, where SotB2 is a Phe whereas SotB is a Tyr (Figure 7D). In addition, in the transport cavities of the two transporters, compared with the total overlap of the positions of amino acids such as L317, Q320, M335, E338, F342, and V222, the positions of the four amino acids N26, E29, F30, and F226 were all significantly moved down in SotB2 compared with SotB (Figure 7D), which make the pocket space of SotB2 larger, and this may explain why SotB2 can transport disaccharides such as melibiose, while SotB cannot. The overall size of the transport cavities of SotB, SotB2, and MdfA also indicate to this point, compared with SotB (width × length ≈ 6 × 9 Å), SotB2 (width × length ≈ 8 × 14 Å) and MdfA (width × length ≈ 9 × 16 Å) have larger binding cavities, which enables them to selectively transport larger compounds based on their overall size (Figures 7F–H).

## Discussion

The major facilitator superfamily (MFS) transporters often contain several conserved motifs in addition to the 12-TM helix overall structure. Among them, Motif A and Motif B are the most common. Mutagenesis analysis have repeatedly shown that Motif A and Motif B are essential for the transport activity in many MFS transporters (Yamaguchi et al., 1992; Jessen et al., 1995; Kimura et al., 1998; Bannam et al., 2004). In SotB, Motif A positions Loop 5–6. Motif B site is not present in SotB. Motif C site is also called the antiporter motif which is located on TM5 of SotB. The function of several conserved amino acids in SotB were also investigated in this study by the L-arabinose inhibition assays (Supplementary Figure S2). The variants (G153A, G153E, G157E, and L377A) were expressed respectively, and mutations at these sites did not affect the expression of the target protein (Supplementary Figure S8). The L-arabinose inhibition assay results show that the transport activities of G153E and G157E variants were significantly decreased compared with wild-type SotB protein and G153A variant (Figure 4B), which is consistent with the study of Ginn et al. (2000), indicating that glycines at the Motif C site may participate in the formation of a pocket, which devoids of side chains, and such a pocket may make the conformation of protein more stable and thus facilitates the efflux of the substrate (Varela et al., 1995). Besides, amino acids of Motif C may also play important roles in linking proton translocation to the antiporter (Paulsen and Skurray, 1993). Taken together, our studies indicate that the conserved residues G153 and G157 of motif C in SotB are important for SotB-mediated L-arabinose transport.

**Figure 8:**
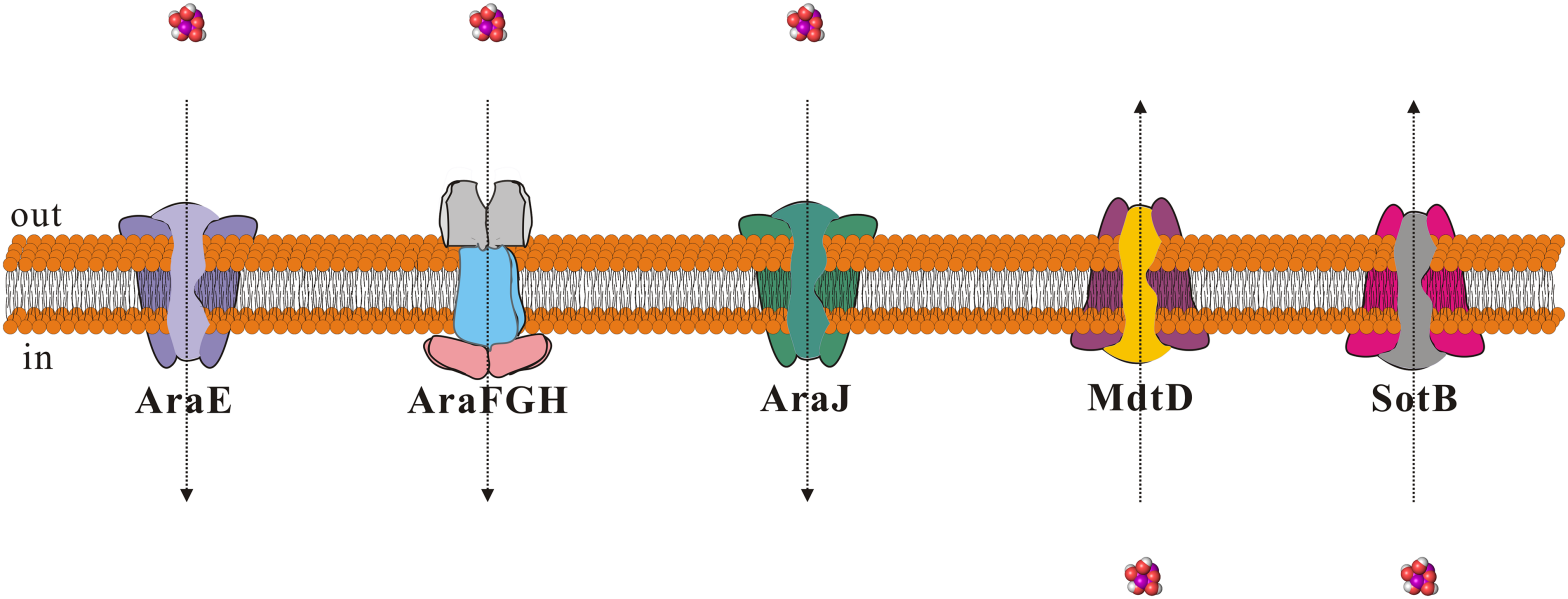
Schematic diagram of proteins that can mediate L-arabinose transport in *Escherichia coli*. The small molecule L-arabinose is shown as purple sphere. Direction of entry and exit of L-arabinose is shown using dotted arrows.

To explore the transport capacity of SotB to L-arabinose, our study first showed that SotB can indeed mediate the export of L-arabinose of *E. coli* directly by using the cell-based radiotracer uptake assay (Figure 1C). On this basis, the binding affinity between SotB and L-arabinose was measured by SPR. The SPR result shows that the affinity of L-arabinose with SotB is 4.1 mM (Figure 3D), which belongs to a weak binding. This coincides the fact that MFS transporters often bind their substrates weakly (high μM to mM range) so that the substrates can be transported across membranes at physiologically relevant concentrations (Lewinson et al., 2006; Fluman et al., 2009; Mueckler and Thorens, 2013; Kaback and Guan, 2019; Minhas and Newstead, 2019). Several transporters that can transport L-arabinose have been found in *E. coli* (Figure 8). AraE encodes a low-affinity L-arabinose transporter, whereas AraFGH encodes a high-affinity L-arabinose transporter system (Stoner and Schleif, 1983; Hendrickson et al., 1992; Koita and Rao, 2012; Ranade and He, 2021). AraJ and MdtD belong to the MFS family (Stoner and Schleif, 1983; Hendrickson et al., 1992; Koita and Rao, 2012; Ranade and He, 2021), and their sequence similarity with SotB are 22.4% and 16.2%, respectively (Supplementary Table S3).

To identify the key amino acids involved in the L-arabinose transport, a structural analysis of the SotB-L-arabinose complex was performed by docking the L-arabinose molecule into the structure of SotB (Figure 3A). In our docking model, E29, H115, W119, S339, and N343 can form hydrogen bonds with L-arabinose (Figure 3B). In the transport cavity of SotB, E29 from TM1 and H115 from TM4 may exist as protonated residues (Figures 3B,C). The L-arabinose inhibition assay results show the mutation from E29 to A29 and from H115 to A115 all significantly reduced the transport activity of SotB (Figure 4A). The binding affinity between E29A variant and L-arabinose is 5.1 mM (Figure 3E), which is almost unchanged compared with the wild-type SotB (Figure 3D), and this result indicate that E29 does not participate in the substrate recognition process. However, the binding affinity of H115A variant with L-arabinose decreases 3.2-fold compared with the binding affinity between wild-type SotB and L-arabinose (Figures 3D,F). Based on the above results, we believe that E29 is more likely to be responsible for protonation, whereas H115 is important in the substrate recognition process. Similarly, by analyzing the results of SPR (Figures 3G-I) and the L-arabinose inhibition assays (Figure 4A), in the SotB-mediated L-arabinose transport process, W119 and S339 are involved in the substrate efflux, and N343 is involved in the substrate recognition.

To better understand the transport relationship between SotB and IPTG, the binding affinities of IPTG with SotB/SotB variants were measured by SPR. The SPR results show that the binding affinity of SotB to IPTG (10.2 mM) is significantly lower than its affinity to L-arabinose (4.1 mM; Figures 3D, 5A). Our SPR results show all the five SotB variants (E29A, H115A, W119A, S339A, and N343A) do not affect the binding between the transporter and IPTG (Figures 5B–F). In the export cavity of SotB, F30, Y226, and F342 can make contacts with IPTG’s hydrophobic tail through van der Waals interactions (Figure 5G). These interactions may also stablize IPTG to some extent and thus minimizing the influence of the alanine mutation on the binding of IPTG to SotB/SotB variants. Even the binding affinities between SotB/SotB variants and IPTG were measured successfully in this study, transport needs to be further assessed. Our experimental results show that SotB can directly export L-arabinose (Figure 1C), but whether SotB can export IPTG remains to be verified by relevant experiments.

The metabolic intermediates of L-arabinose are toxic, which may be the main reason for the efflux of L-arabinose by SotB (Figures 2A,B; Koita and Rao, 2012). To better understand the binding affinity characteristic between SotB and the substrate L-arabinose, the binding affinities of a series of L-arabinose analogs to SotB were measured. The SPR results show that the binding affinities of D-fructose, D-glucose, and D-mannose to SotB are 6.6, 2.6, and 3.1 mM (Figures 6A,B,D), respectively, which are almost the same as the binding affinity of L-arabinose to SotB (Figure 3D). To our surprise, the binding affinity of D-xylose to SotB is 0.47 mM (Figure 6C), which is 8.7-fold higher than the affinity of L-arabinose to SotB. So far, there is no evidence that SotB can transport D-xylose. The above SPR information (Figure 6C), combined with the related L-arabinose inhibition assay results (Figures 4C,D), we think D-xylose may be an inhibitor of the L-arabinose transport process mediated by SotB. Notably, according to our SPR results, the binding of IPTG/L-arabinose/L-arabinose analogs to SotB/SotB variants are all at low affinities (mM range), these results combined with related studies indicate that MFS family proteins like SotB may naturally be in the low binding affinity with their substrate(s) (Dang et al., 2010; Ethayathulla et al., 2014; Heng et al., 2015).

To elucidate the difference in substrate transport between SotB and its homologous protein SotB2, the three-dimensional (3D) structure of SotB2 was modeled by AlphaFold 2 program (Figures 7A–C), and made a detailed comparative analysis with the structure of SotB. The crystal structure of SotB and the AlphaFold 2 predicted structure of SotB2 are directly compared. The overall structures of SotB and predicted SotB2 superimposed very well except the loops and N- and C-terminal (Figure 7E). Then, by comparing the composition and size of SotB and SotB2 transport cavities (Figure 7D), our study successfully illustrated that compared with SotB, SotB2 has a larger transport cavity, which enables it to transport larger substrates such as lactose and melibiose.

**Figure 9:**
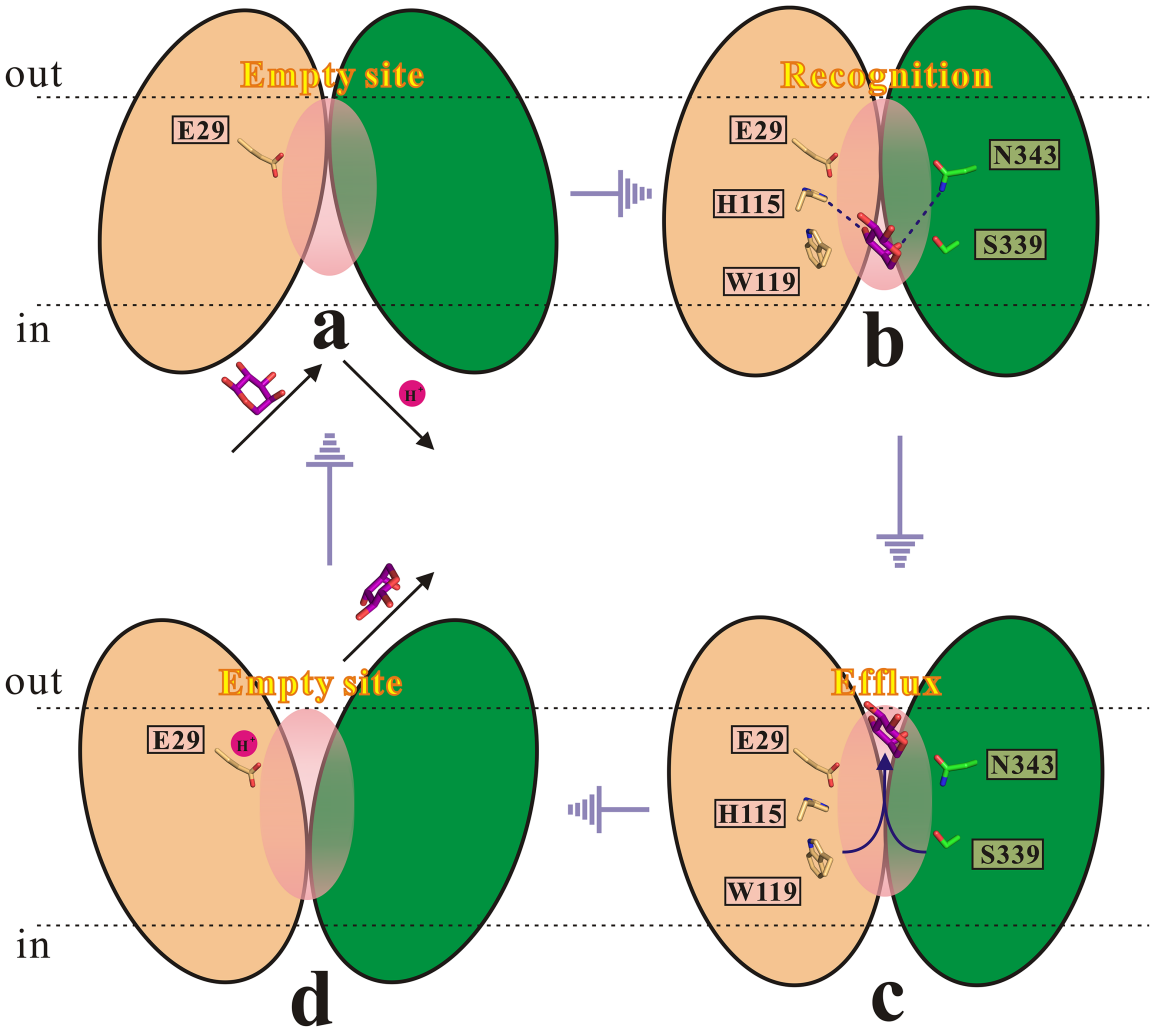
Proposed transport cycle of SotB. The N-terminal and C-terminal domain of SotB are shown as wheat and green color cartoon, respectively. Key functionally important residues (E29, H115, W119, S339, and N343) are shown as sticks. The L-arabinose molecule is shown as purple sticks.

Based on above findings, combined with related studies about the rocker-switch mechanism (Abramson et al., 2003; Huang et al., 2003; Shi, 2013; Fukuda et al., 2015; Yan, 2015; Drew and Boudker, 2016; Quistgaard et al., 2016; Wu et al., 2019), a mechanism for the SotB mediated L-arabinose transport cycle is proposed in this study (Figure 9). In antiporters, substrate binding and protonation are often found to compete with each other (Fluman et al., 2012). Substrate binding-induced deprotonation is thought to trigger the conformational change from inward-facing to outward-facing. First, in the inward-open state (a) and before substrate loading, SotB E29 remains protonated. The inward-open state is ready for substrate L-arabinose loading-triggered deprotonation. A H^+^ is likely to be released from E29 to the cytosol upon L-arabinose binding. In this process, H115 and N343 are responsible for L-arabinose recognition. Second, in the inward-occluded state (b), L-arabinose binding triggers a conformational switch that exposes the L-arabinose binding pocket to the extracellular side (periplasm). Third, in the outward-occluded state (c), L-arabinose is released to the periplasmic side. W119 and S339 are directly involved in this efflux process. Fourth, in the outward-open state (d), after L-arabinose molecule is released, E29 is protonated again. As L-arabinose binds to SotB from the inner side of the cell and protonation of E29 provides the driving force for the C_Out_-to-C_In_ conformational change, so the whole transport process finally from state (d) returns to the initial state (a).

Although some progress in the function and mechanism of SotB have made in this study, it remains some questions unclear. For SotB, it has not been determined whether it can directly transport IPTG. Besides, although a relatively accurate transport model of SotB based on our experimental results is proposed, the order of protonation/deprotonation and the connection with L-arabinose binding and release has not yet been established in detail, so confirming this hypothesis will require further experimental validation. To fully understand the transport mechanism of MFS Mdr transporters like SotB, future work will be required to define and verify details of related mechanisms and involve techniques such as fluorescence resonance energy transfer (FRET), nuclear magnetic resonance (NMR), electron paramagnetic resonance (EPR) spectroscopy and related computational methods (Puthenveetil and Vinogradova, 2019; Bonucci et al., 2020; Jiang et al., 2020; Bartels et al., 2021).

## Data availability statement

The original contributions presented in the study are included in the article/Supplementary material, further inquiries can be directed to the corresponding authors.

## Author contributions

ZZ and GZ conceived the project and designed experiments. GZ performed all the experiments and related structural analysis in this study and wrote the manuscript. ZZ and CD revised the manuscript and supervised the project. All authors contributed to the article and approved the submitted version.

## Funding

This work was supported by grants from the National Natural Science Foundation of China (31970052).

## Conflict of interest

The authors declare that the research was conducted in the absence of any commercial or financial relationships that could be construed as a potential conflict of interest.

## Publisher’s note

All claims expressed in this article are solely those of the authors and do not necessarily represent those of their affiliated organizations, or those of the publisher, the editors and the reviewers. Any product that may be evaluated in this article, or claim that may be made by its manufacturer, is not guaranteed or endorsed by the publisher.

## Footnotes

^1^ http://www.tcdb.org

^2^ https://benchling.com/editor

^3^ https://espript.ibcp.fr/ESPript/cgi-bin/ESPript.cgi

^4^ https://colab.research.google.com/github/sokrypton/ColabFold/blob/main/AlphaFold2.ipynb#scrollTo=KK7X9T44pWb7

^5^ https://saves.mbi.ucla.edu/

^6^ https://pymol.org/2/

^7^ https://pymol.org/2/
