# Supplementary material for: Mutagenesis and functional analysis of SotB: A multidrug transporter of the major facilitator superfamily from Escherichia coli
Source: Front Microbiol. 2022 Oct 28;13:1024639. doi: 10.3389/fmicb.2022.1024639 (PMC9650428; doi:10.3389/fmicb.2022.1024639)
Supplement: Supplementary file 1 [file Data_Sheet_1.doc]

Supplementary Material

# Supplementary Tables

| **Supplementary Table 1.** **List of bacterial strains, plasmids and primers used in the study.** | | |
| --- | --- | --- |
| **Strains/plasmids/ primers** | **Description** | **Sourse** |
| **Strains**  ***E. coli*** | | |
| DH10B | F^-^,*mcrA*, Δ(*mrr-hsd*RMS*-mcr*BC), φ80d, *lac*ZΔM15, Δ*lac*X74, *deo*R, *rec*A, *lend*A, *lara*Δ139, D(*ara*, *leu*)7697, *gal*U, *gal*K, λ^-^, *rps*L, *nup*G | Gibco-BRL |
| MG1655 | F^-^, lambda^-^, ilvG^-^, rfb^-^50, rph^-^1 | Lab storage |
| MG1655::pEcCas | MG1655 strain containing pEcCas plasmid | Lab storage |
| C43(DE3) | F^-^, *ompT*, *hsdS_B_*(*r_B_^-^m_B_^-^*), gal *dcm* (DE3) | NEB |
| **Plasmids** | | |
| pET28a | *neo*, rep^pMB1^, T7 promoter, Km^r^ | Novagen |
| pET28a/*sotB* | pET28a derivative containing *sotB* gene | This study |
| pET28a/*sotB_E29A_* | pET28a derivative containing *sotB_E29A_* gene | This study |
| pET28a/*sotB_H115A_* | pET28a derivative containing *sotB_H115A_* gene | This study |
| pET28a/*sotB_W119A_* | pET28a derivative containing *sotB_W119A_* gene | This study |
| pET28a/*sotB_G153A_* | pET28a derivative containing *sotB_G153A_* gene | This study |
| pET28a/*sotB_G153E_* | pET28a derivative containing *sotB_G153E_* gene | This study |
| pET28a/*sotB_G157E_* | pET28a derivative containing *sotB_G157E_* gene | This study |
| pET28a/*sotB_S339A_* | pET28a derivative containing *sotB_S339A_* gene | This study |
| pET28a/*sotB_N343A_* | pET28a derivative containing *sotB_N343A_* gene | This study |
| pET28a/*sotB_L377A_* | pET28a derivative containing *sotB_L377A_* gene | This study |
| pET28a/*lapC* | pET28a derivative containing *lapC* gene | This study |
| pEcCas | sacB, P*_rhaB_*-sgRNA-pMB1, rep^pSC101^, Km^r^ | (Li et al., 2021) |
| pEcgRNA | *ccdB,* Sm^r^ | (Li et al., 2021) |
| pEcgRNA/*sotB* | Derived from pEcgRNA, target *sotB* in *E. coli* MG1655 | This study |
| **Primers (5’→3’)** | | |
| SotB-1F | CTTTAAGAAGGAGATATACCATGACAACAAACACTGTTTCCCGCAAAGTG |  |
| SotB-1R | GTGGTGGTGGTGGTGGTGTTGCGTCTGTTCTTCGAGTG |  |
| SotB-2F | CACCACCACCACCACCACTGAGATC |  |
| SotB-2R | GGTATATCTCCTTCTTAAAGTTAAACAAAATTATTTCTAGAG |  |
| SotB_E29A_-1F | CAACACCACCGCATTTGTCCCTGTTGGCCTGCTCTCTG |  |
| SotB_E29A_-1R | GGGACAAATGCGGTGGTGTTGAAGATGAAGGCGGCGAC |  |
| SotB_H115A_-1F | CTTTTGCAGCAGCGATTTTCTGGTCGATTACGGCGT |  |
| SotB_H115A_-1R | GAAAATCGCTGCTGCAAAAGCCACACCAATGCGACTG |  |
| SotB_W119A_-1F | GCGATTTTCGCGTCGATTACGGCGTCTCTGGC |  |
| SotB_W119A_-1R | CGTAATCGACGCGAAAATCGCATGTGCAAAAGCCACAC |  |
| SotB_G153A_-1F | GATGGTCTTAGCATTACCTCTCGGGCGCATTGTGGGCCAG |  |
| SotB_G153A_-1R | GAGGTAATGCTAAGACCATCGCCAGTGCTGTACCGGTG |  |
| SotB_G153E_-1F | GATGGTCTTAGAGTTACCTCTCGGGCGCATTGTGGGCCAG |  |
| SotB_G153E_-1R | GAGGTAACTCTAAGACCATCGCCAGTGCTGTACCGGTG |  |
| SotB_G157E_-1F | TACCTCTCGAACGCATTGTGGGCCAGTATTTCGGTTG |  |
| SotB_G157E_-1R | CACAATGCGTTCGAGAGGTAAACCTAAGACCATCGCCAG |  |
| SotB_S339A_-1F | GATGGCGCTATTCGCCGGCATATTTAATATTGGAATCGG |  |
| SotB_S339A_-1R | AATATGCCGGCGAATAGCGCCATCGCGACG |  |
| SotB_N343A_-1F | GCATATTTGCAATTGGAATCGGGGCGGGTGCGTTGG |  |
| SotB_N343A_-1R | GATTCCAATTGCAAATATGCCGGAGAATAGCGCCATC |  |
| SotB_L377A_-1F | TTGCCGCGGCAATTTGGTCAATCATTATATTTCGCCGCTG |  |
| SotB_L377A_-1R | TGACCAAATTGCCGCGGCAAAAGCAGGCACCGCGCCCAC |  |
| LapC-1F | ACTTTAAGAAGGAGATATACCATGGTAACTCATCGTCAGCGCTAC |  |
| LapC-1R | GTGGTGGTGGTGGTGGTGGTGTCAGTTAGCGATAAAACGCTTCTC |  |
| LapC-2F | CACCACCACCACCACCACTGAGATC |  |
| LapC-2R | GGTATATCTCCTTCTTAAAGTTAAACAAAATTATTTCTAGAG |  |
| gRNA-F | TAGTCGTTAATTTGGTCAATCATT |  |
| gRNA-R | AAACAATGATTGACCAAATTAACG |  |
| Donour DNA-1F | TCTAACAAACATGTACTAC |  |
| Donour DNA-1R | TGGGCCTTTCAACTAAACAGATTATCGGGTGAGTTG |  |
| Donour DNA-2F | TAGTTGAAAGGCCCATTCG |  |
| Donour DNA-2R | TGATGGATACATTTGGCATTTC |  |
| Very-1F | CCAGGAATGGAAGACACAAAG |  |
| Very-1R | CTGATTAACCATATGGATTAAGCG |  |

Abbreviations: Km^r^, kanamycin resistance; Sm^r^, [Spectinomycin](https://www.chembk.com/en/chem/spectinomycin) resistance; All primers were synthesized by Tsingke Biotechnology Co., Ltd (Beijing, China).

**Supplementary Table 2. Sequence similarity analysis of SotB, EmrD, LmrP, MdfA and YajR.**

| % ID | SotB | EmrD | LmrP | MdfA | YajR |
| --- | --- | --- | --- | --- | --- |
| SotB | - | 16.1 | 16.4 | 13.4 | 13.8 |
| EmrD | 16.1 | - | 17.7 | 20.2 | 16.9 |
| LmrP | 16.4 | 17.7 | - | 16.7 | 15.9 |
| MdfA | 13.4 | 20.2 | 16.7 | - | 16.8 |
| YajR | 13.8 | 16.9 | 15.9 | 16.8 | - |

***Supplementary Table 3. Sequence similarity analysis of L-arabinose transporters in E. coli.***

| % ID | SotB | SotA | AraJ | MdtD |
| --- | --- | --- | --- | --- |
| SotB | - | 16.6 | 22.4 | 16.2 |
| SotA | 16.6 | - | 15.1 | 12.3 |
| AraJ | 22.4 | 15.1 | - | 16.5 |
| MdtD | 16.2 | 12.3 | 16.5 | - |

# Supplementary Figures


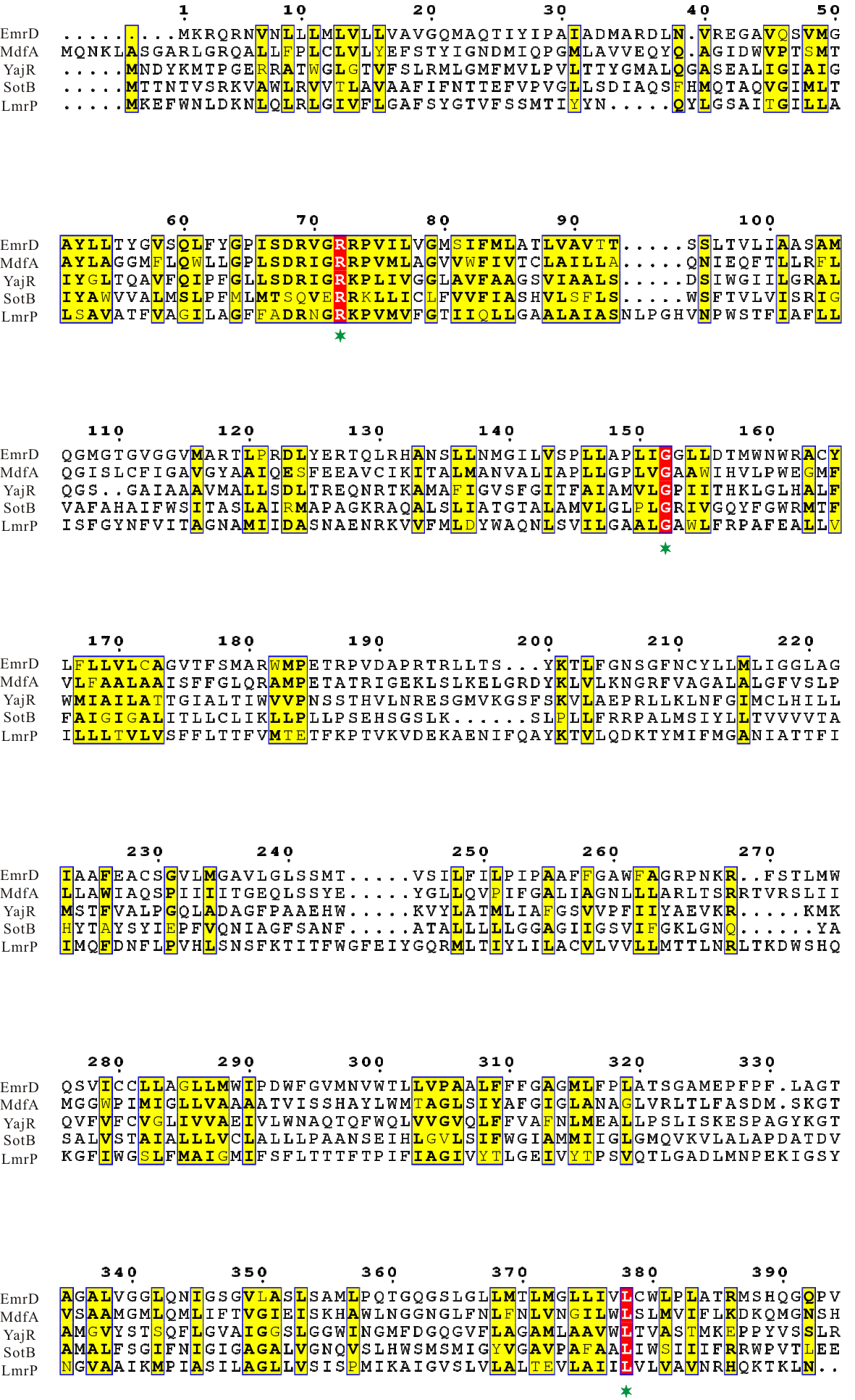


**Supplementary Figure 1. The protein sequence alignment of MFS Mdr transporters SotB, EmrD, YajR, MdfA and LmrP.** The overall sequence similarity of these five MFS Mdr transporters is very low, and R78, G157 and L377 in SotB are fully conserved. The conserved sites are highlighted with green asterisk.


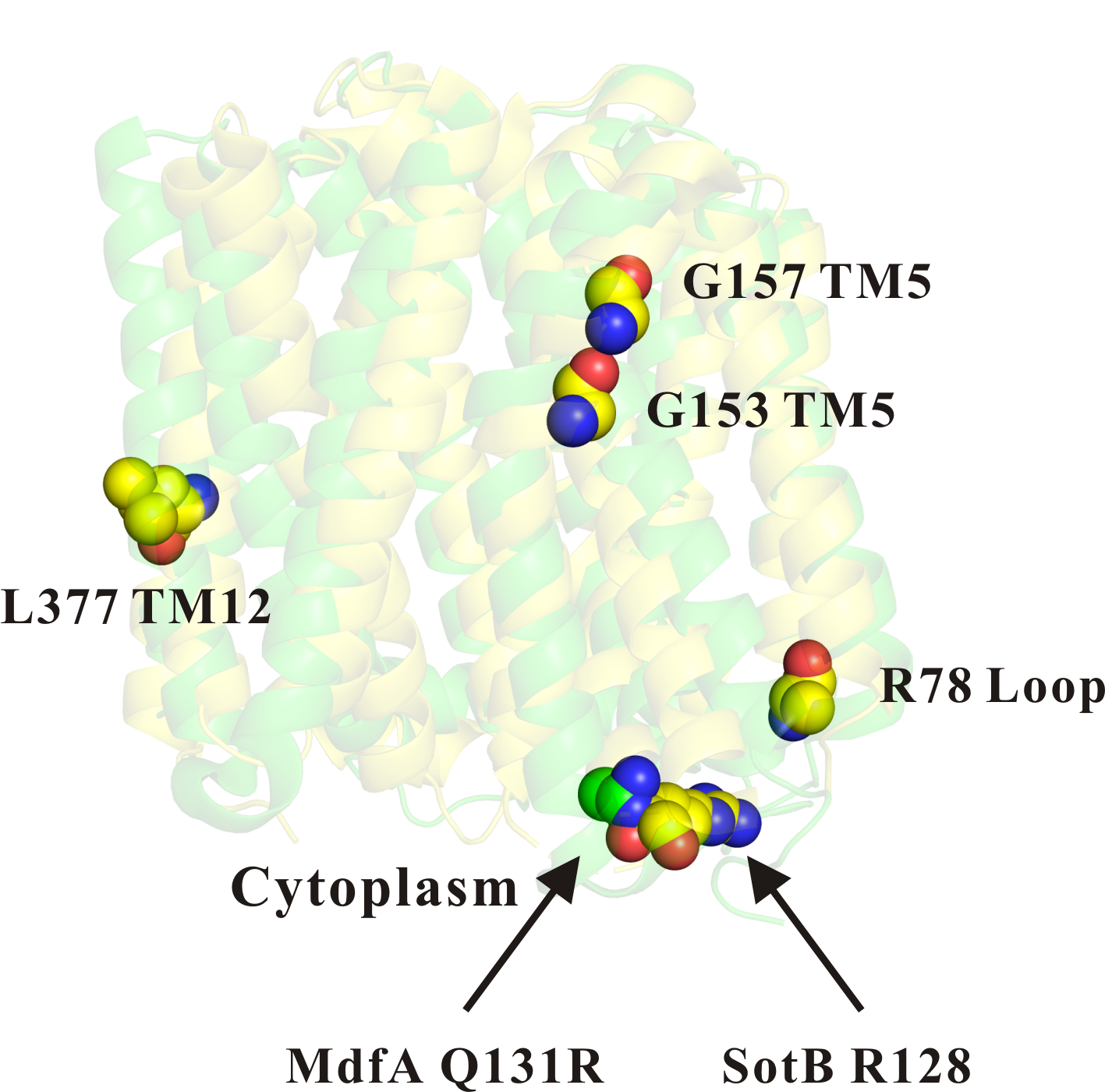


**Supplementary Figure 2. The superimposition of SotB (PDB ID: 6KKI) and MdfA (PDB ID: 4ZP0).** The sequence similarity of SotB (yellow cartoon) and MdfA (green cartoon) is 13.4%, and the r.m.s.d. of structural comparison between them is 2.8 Å over 359 residues. The conserved amino acids in SotB (R78, G153, G157, and L337) and amino acid R131 in MdfA Q131R are shown as yellow and green spheres, respectively.


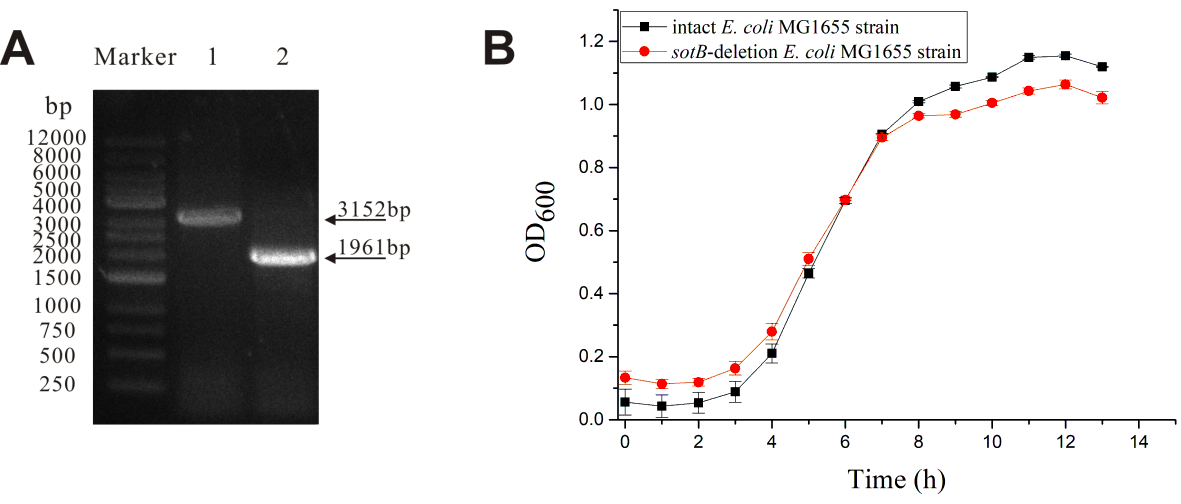


**Supplementary Figure 3.** **Growth curves of intact *E. coli* MG1655 strain and *sotB*-deletion *E. coli* MG1655 strain in the presence of 1 mM L-arabinose. (A)** PCR identification of the in-frame deletion of *sotB* gene in *E. coli* MG1655. Lane 1 refers to the PCR identification result of *E. coli* MG1655 with *sotB* gene, Lane 2 refers to the PCR identification result of *E. coli* MG1655 without *sotB* gene. (B) In the presence of 1 mM L-arabinose, the intact MG1655 grew better compared to *sotB*-deletion MG1655, and this result suggest that the deletion of *sotB* gene will prevent the efflux of L-arabinose making the strain more sensitive to L-arabinose.


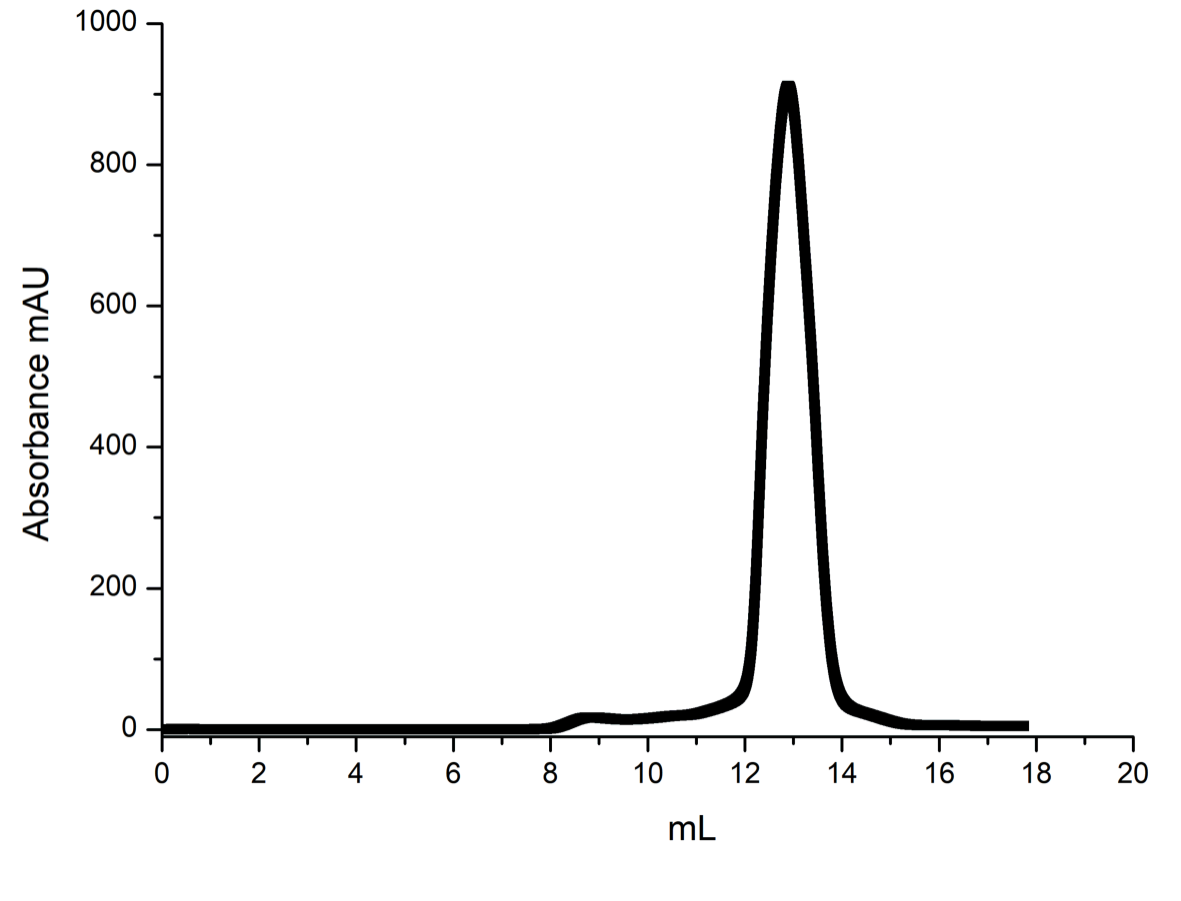


**Supplementary Figure 4. Size exclusion chromatography of SotB.** SotB (MW: 42.5 kDa) elutes at 13 mL on a Superdex 200 Increase 10/300 GL column (Cytiva), corresponding to an apparent molecular weight of 440 kDa, the peak position of SotB protein is advanced due to the wrapping of detergent DDM.


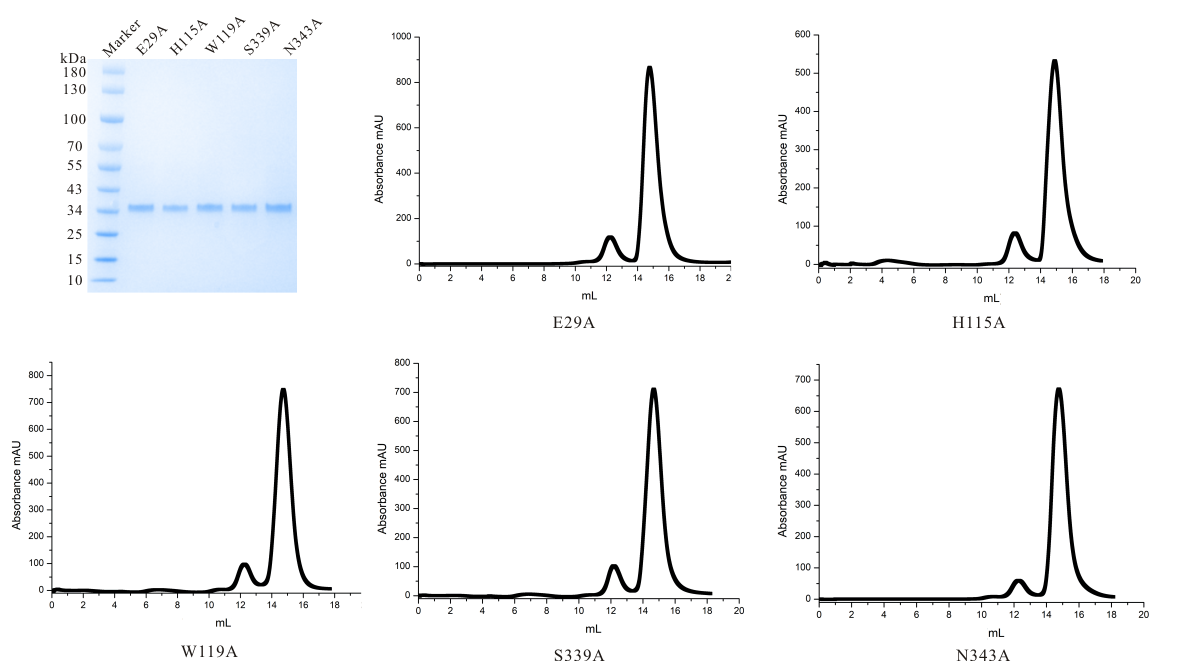


**Supplementary Figure 5. SDS-PAGE and size exclusion chromatography analysis of SotB variants.** These results show that these five SotB variants (E29A, H115A, W119A, S339A and N343A) are all expressed normally.


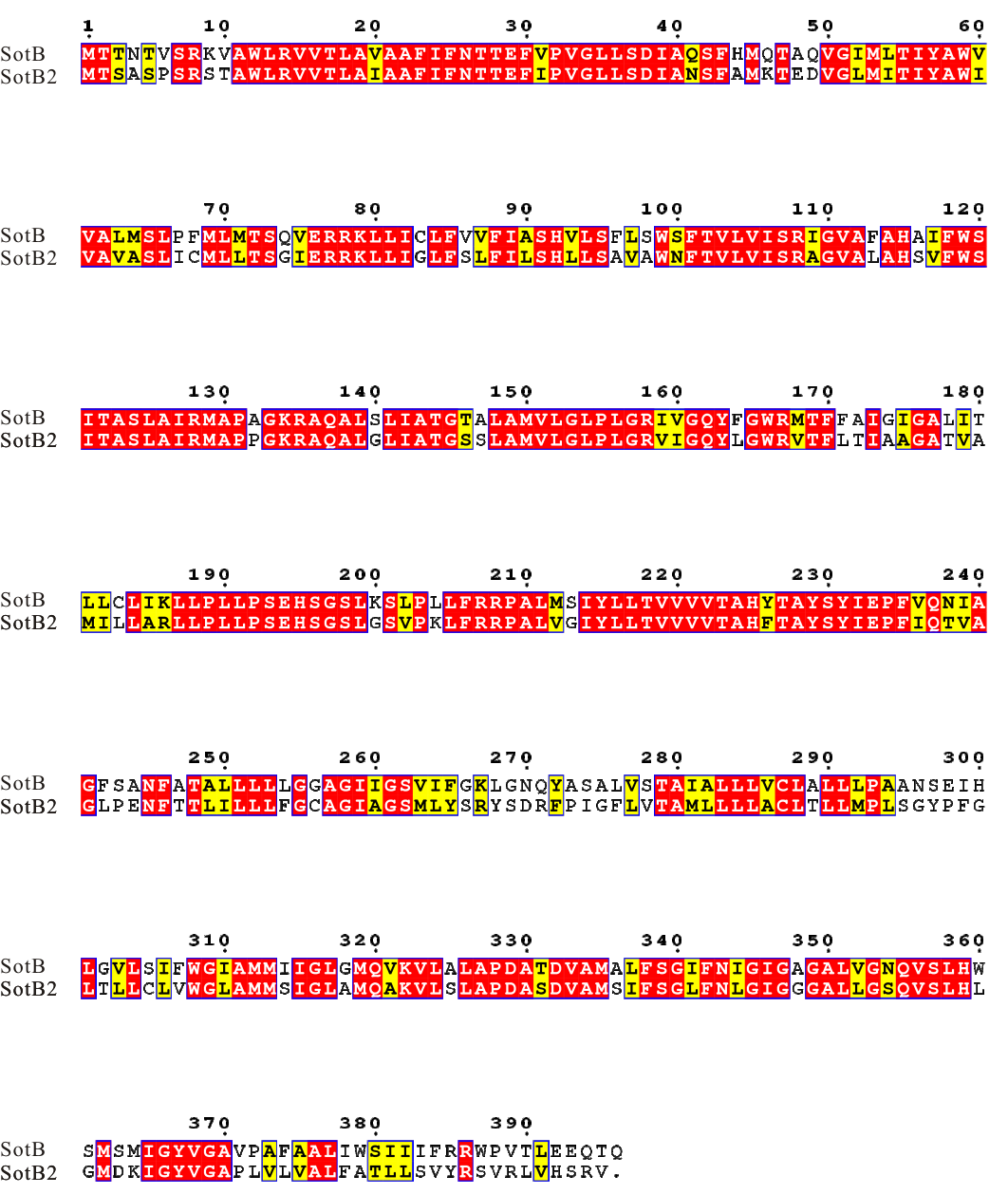


**Supplementary Figure 6. Bioinformatic analysis of SotB with its homologs protein SotB2.** SotB is from *Escherichia coli,* and SotB2 is from *Erwinia chrysanthemi.* The protein sequence similarity between SotB and SotB2 is 62.8%.


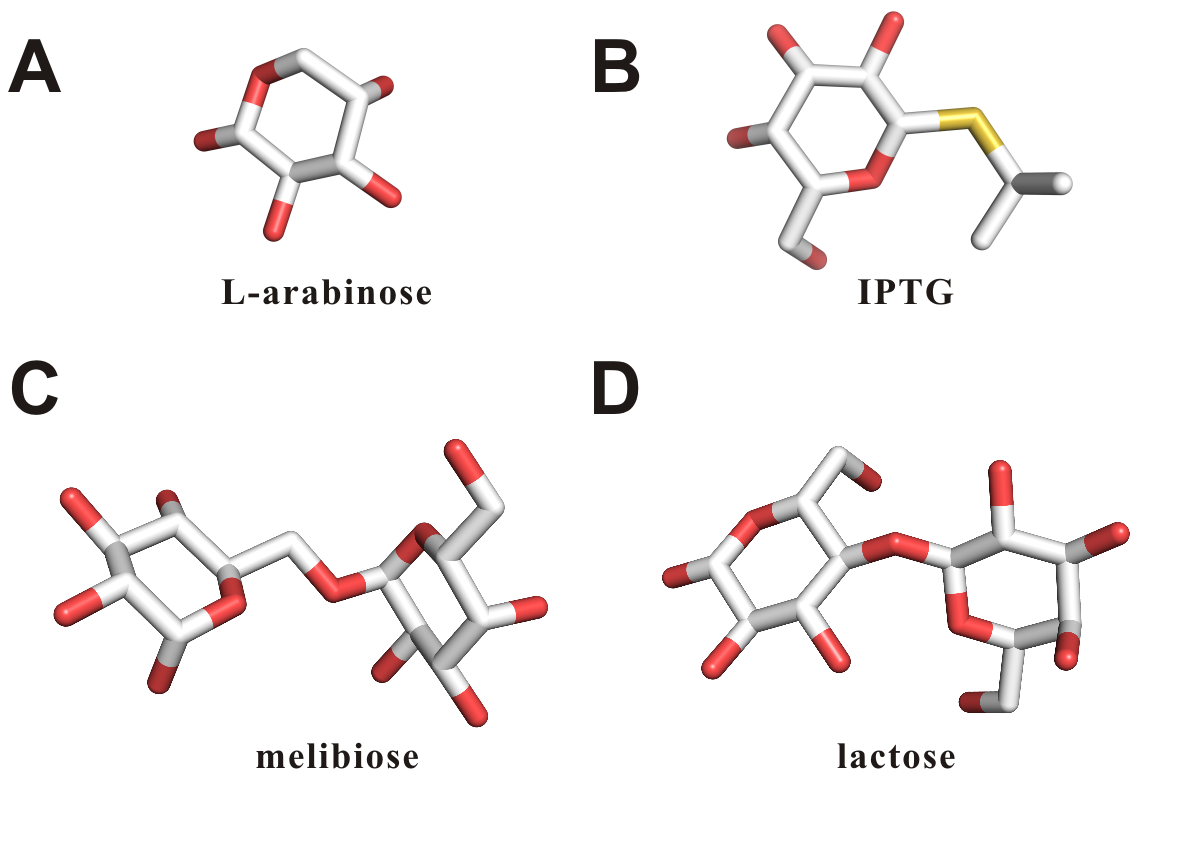


**Supplementary Figure 7. Substrates of SotB and SotB2.** (A) The transport substrate of SotB, L-arabinose. (B) The potential transport substrate of SotB, IPTG. (C) The transport substrate of SotB2, melibiose. (D) The transport substrate of SotB2, lactose. All the substrates are shown as gray sticks. Oxygen atoms are in red, and sulfur atom is in yellow.


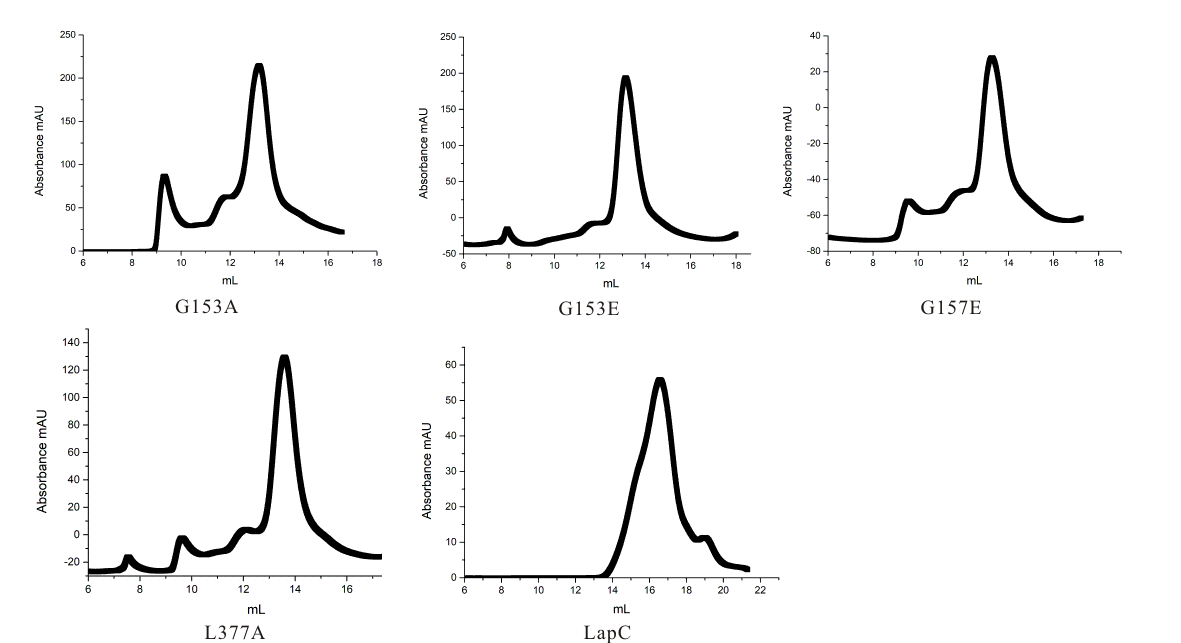


**Supplementary Figure 8. Size exclusion chromatography analysis of SotB variants and LapC.** The results show that these SotB variants (G153A, G153E, G157E, and L337A) and LapC are all expressed normally.
